# Multifactorial conceptual model of cancer-related accelerated aging

**DOI:** 10.1038/s41514-025-00328-8

**Published:** 2026-01-16

**Authors:** Lisa Morse, Sandra Weiss, Christine S. Ritchie, Melisa L. Wong, Thomas Hoffmann, Margaret Wallhagen, Christine Miaskowski

**Affiliations:** 1 School of Nursing, University of California, San Francisco, CA USA; 2 Department of Veterans Affairs, Veterans Health Administration, San Francisco, CA USA; 3 School of Medicine, Harvard Medical School, Boston, MA USA; 4 Division of Palliative Care and Geriatric Medicine, Massachusetts General Hospital, Morgan Institute, Boston, MA USA; 5 Division of Research, Kaiser Permanente Medical Group, Oakland, CA USA; 6 School of Medicine, University of California, San Francisco, CA USA

**Keywords:** Cancer, Health care, Oncology, Risk factors

## Abstract

Evidence suggests that cancer-related accelerated aging contributes to an earlier onset of chronic diseases; persistent symptoms; and decrements in patients’ quality of life. This review presents the Multifactorial Model of Cancer-related Accelerated Aging (MMCRAA), a conceptual framework that is grounded in Life Course Theory and supported by empiric evidence. The model includes six inter-related concepts: person, behavioral, biological, treatment, symptom, and life course factors. The MMCRAA can be used by clinicians and researchers to identify patients at increased risk for cancer-related accelerated aging; guide personalized treatment planning; and inform the development of interventions and research.

## Introduction

While advances in cancer detection and treatment have led to reductions in cancer mortality^1^, these increases in life expectancy can be accompanied by an earlier onset of chronic disease^2^, persistent unrelieved symptoms^3^, and diminished quality of life^4^. For example, compared to the general population, some patients with cancer experience an earlier onset of multi-morbidity^2,5–7^, frailty^8^, cognitive impairment^9^, and functional decline^10^. A growing body of evidence suggests that cancer-related accelerated aging contributes to these health burdens^11–13^.

**Fig. 1:**
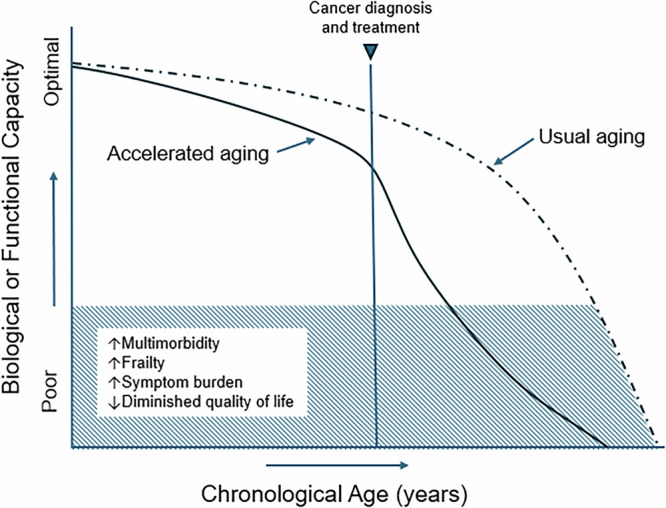
Hypothesized aging trajectories in patients with cancer receiving treatment. This figure depicts the relationship between biological or functional capacity and chronological age in individuals experiencing usual versus accelerated aging. The solid line illustrates an accelerated aging trajectory, marked by an earlier onset and longer duration of health burdens such as multimorbidity, frailty, persistent symptoms, and reduced quality of life. In addition, depending on various early life exposures and genetic risk factors, the accelerated aging trajectory may be present before cancer diagnosis and treatment. The dashed line represents a usual aging trajectory, with a shorter period of health decline occurring closer to the end of life. (Adapted from Guida et al.).

Accelerated aging is characterized by physiological (i.e., reduced ability to maintain homeostasis) and/or functional decline that occurs earlier than expected for a patient’s chronological age^14^. Estimates of biological or functional age, terms often used interchangeably, compare a patient’s biological parameter(s) or clinical assessment(s) to norms for the general population at a specific chronological age. Patients with a biological or functional age that exceeds their chronological age are considered to be accelerated in their aging (see Fig. 1). While correlated with chronological age, estimates of biological and/or functional age more accurately predict inter-individual variability in the severity of functional decline, increased risk for mortality, and the occurrence of diseases associated with aging^15^.

**Fig. 2:**
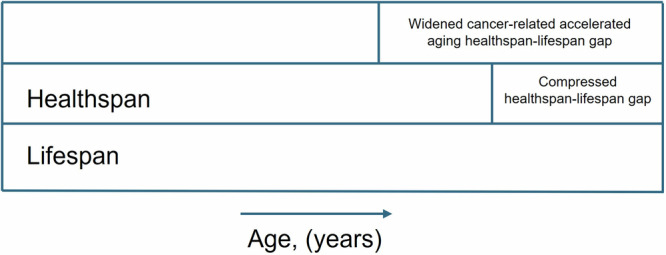
The healthspan lifespan gap in patients with cancer. The figure represents a widened or compressed healthspan lifespan gap in patients with cancer and survivors of cancer. The healthspan is the number of years lived in good health. The lifespan is the total number of years lived. The healthspan-lifespan gap is characterized by the presence of chronic conditions, persistent unrelieved symptoms, and poorer quality of life. Cancer-related accelerated aging is hypothesized to widen the gap between the healthspan and lifespan.

Compression of morbidity is a concept that refers to extending the amount of time in good health (i.e., healthspan) by delaying the time spent in poor health to a shorter duration at the end of life^16^. As shown in Fig. 2, cancer-related accelerated aging appears to widen the gap between the healthspan and lifespan. In fact, depending on various multifactorial factors (e.g., sociodemographic context^17^, chronological age^18^), patients with cancer can lose between 13 and 17 years of quality-adjusted life years due to their illness^19^. Importantly, the global burden of cancer is growing^17^. As the number of cancer survivors in the United States continues to increase^20^, the identification of risk factors associated with cancer-related accelerated aging is essential to be able to compress morbidity, improve quality of life, and prevent the earlier onset of poor health outcomes in these individuals.

The identification of risk factors associated with cancer-related accelerated aging is hindered by several methodological and conceptual issues. In terms of methodological constraints, existing measures used to estimate biological and/or functional age require refinements^21,22^. In fact, no gold standard exists to estimate accelerated aging. As a result, various approaches are used across studies, making it difficult to compare and contrast findings^21,23^.

In terms of conceptual challenges, while clinical manifestations of accelerated aging in the general population have been described^21^, the cancer-related accelerated aging phenotype is not well characterized. For example, an interdisciplinary panel of experts proposed six clinical signs and symptoms (i.e., fatigue, poor sleep, depressed mood, lack of motivation, subjective memory complaints, poor exercise tolerance) that occur as a result of the biological mechanisms associated with cellular decline and accelerated aging in the general population^21^. However, in patients with cancer, distinct signs and symptoms warrant consideration. For example, the presence of frailty^24^ (i.e., sarcopenia, weakness, slow walking speed, low energy, fatigue, and exhaustion) and cancer-related symptoms (i.e., fatigue^25,26^, pain^26^, sleep disturbance^26^, peripheral neuropathy^27^, cognitive changes^26^, depression^26^, and anxiety^26^) are associated with accelerated aging. Without a clearly defined cancer-related accelerated aging phenotype, salient risk factors associated with its occurrence are not available to researchers and clinicians.

An equally challenging conceptual issue is the establishment of a standard for healthy aging. Most of the current approaches to determine usual versus pathologic aging take physiologic (e.g., inflammatory markers), omic (e.g., Deoxyribonucleic Acid (i.e., DNA) methylation), or deficit (e.g., number of comorbidities, functional decline) perspectives^15^. However, findings from studies of older adults suggest that healthy aging encompasses more than not having a certain number of accumulated chronic conditions. For example, older adults may self-report that they are “aging successfully” despite having chronic medical conditions or physical disability^28,29^. In contrast, poorer self-rated health is associated with increased accelerated aging^30,31^. Equally important, as noted in a review on the domains and measurements of healthy aging^32^, physiological wellness and social engagement are common standards by which healthy aging is assessed. Currently, research on the relationships between/among these various domains and cancer-related accelerated aging is limited.

Several theories of aging propose the mechanisms and factors that influence the heterogeneity in aging phenotypes among the general population. These aging theories primarily fall into programmed or non-programmed categories. According to the programmed theories (e.g., Gene Regulation Theory^33^, Cellular Senescence Telomere Theory^34–36^), the pace of aging depends on pre-programmed biological clocks that regulate human development through distinct stages (i.e., differentiation, growth, maturity, senescence). Non-programmed theories of aging (e.g., Mutation Accumulation Theory^37^, Error Catastrophe Theory^38^, Disposable Soma Theory^39^, and Free Radical Theory^40^) propose that aging is a consequence of unrepaired cellular damage and/or somatic mutations that accumulate from physiological wear and tear over time. Cellular and/or genetic damage that accumulates over time is thought to eventually exceed the body’s capacity to self-repair, leading to an increased risk for functional decline, chronic illnesses, and mortality^41^. Given the known genotoxic and cytotoxic effects of many cancer therapies, as well as the shared processes between aging and those that enable tumorigenesis (i.e., hallmarks of cancer^42^), cancer and its treatment(s) may act synergistically with biological processes involved in aging (i.e., hallmarks of aging^43^) to accelerate biological aging, increase vulnerability to age-related comorbidities, and exacerbate functional decline in patients with cancer.

In recent years, aging is understood to be a process that is shaped by numerous interrelated intrinsic, extrinsic, and stochastic factors that operate across various levels of biological organization^44^. This perspective has underpinnings in the Life Course Theory^45^, which conceptualizes human development as a lifelong process that is influenced by biological, psychological, and social factors. Building on the tenants of the Life Course Theory^45^, the Social Hallmarks of Aging framework suggests that social causes (e.g., low lifetime socioeconomic status, adversity in childhood and adulthood, being a member of a minority group, adverse health behaviors, adverse psychological states) contribute to inter-individual variability in age-related health outcomes^46^. Moreover, the weathering hypothesis offers a related explanation, proposing that an earlier onset of health deterioration results from the cumulative impact of repeated exposures to social and economic adversity and political marginalization^47^. These theories and associated hypothesis are supported by empiric evidence that suggests that demographic^48^, environmental^49^, behavioral^48^, and psychosocial^50^ factors play a role in driving molecular processes that result in accelerated aging phenotypes in patients with cancer.

Considering the breadth of the theoretical and empiric work done to date, cancer-related accelerated aging is likely to be a multifactorial process. A comprehensive model that includes the various determinants of cancer-related accelerated aging across the lifespan is needed to understand the broad range of potential risk factors and mechanisms that underlie variations in oncology patients’ age trajectories. An increased understanding of the determinants of cancer-related accelerated aging will provide directions for future research that aims to identify risk factors and their relative contribution to age-related health outcomes so that effective interventions can be developed and tested to prevent or treat cancer-related accelerated aging.

Previously, Carroll and colleagues proposed the model of biobehavioral modifiers of cancer and accelerated aging^51^. This model highlights the importance of considering treatment exposures and psychosocial and behavioral risk factors in research and clinical interventions aimed at inhibiting the acceleration of biological mechanisms associated with aging and cancer. While informative, this model is limited to modifiable patient-level behaviors (i.e., psychosocial stress, sleep quality, physical activity, weight management, and substance use). Given that patients with cancer live and interact within a broader historic and sociogeographic context, and evidence supports that these factors influence aging trajectories^49,52^, a more comprehensive model that includes a life course perspective is needed. In addition, including non-modifiable risk factors is important so that they can be accounted for when determining the relationships between and among risk factors related to cancer-related accelerated aging.

A life course perspective emphasizes that human development and aging are lifelong processes and that early-life conditions and experiences can have latent effects on health^53,54^. It highlights how life trajectories are shaped by individual choices, structural opportunities and constraints, and the historical time and place in which a person lives^55^. While Manelblatt and colleagues developed a framework that applies a life course perspective to biological aging in patients with cancer^56^, their model lacks these life course constructs.

Therefore, the purpose of this paper is to present the Multifactorial Model of Cancer-related Accelerated Aging (MMCRAA), a conceptual framework that is grounded in theory and supported by empiric evidence. A conceptual framework provides an orienting scheme that illustrates the relationships between and among concepts and a phenomenon of interest. The MMCRAA can inform future research by identifying key risk factors and their relationships with one or more health outcomes^57,58^.

## Model development

### Literature review

The MMCRAA was developed based on a literature review of the factors associated with cancer-related accelerated aging. The search was conducted in PubMed and Google Scholar using the following keywords and/or phrases: accelerated aging OR biological aging AND cancer OR oncology. The reference list of included studies was searched by hand to identify additional relevant studies. Given the paucity of studies on accelerated aging in patients with cancer, the literature review was expanded to include studies of the general population.

### Conceptual organization of the MMCRAA

**Fig. 3:**
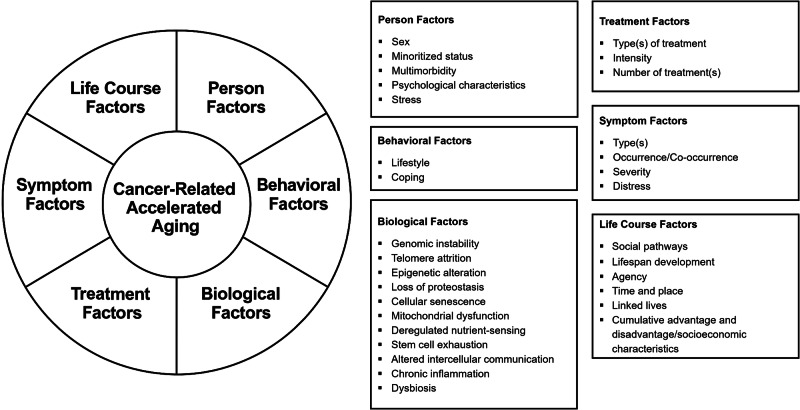
Multifactorial model of cancer-related accelerated aging. Cancer-related accelerated aging is the main construct, represented by the innermost circle in the model. Surrounding this central core is a concentric circle that is partitioned into several sections. Each section represents a distinct concept. The concepts are hypothesized to interact and contribute to cancer-related accelerated aging through complex multi-level pathways that may be unidirectional, bidirectional, or moderated.

Once the factors associated with accelerated aging in patients with cancer were identified, they were organized into broader concepts. A concept represents a set of similar attributes and/or characteristics associated with the phenomenon of interest^57^. As illustrated in Fig. 3, cancer-related accelerated aging is the main construct, represented by the innermost circle in the model. Surrounding this central core is a concentric circle that is partitioned into several sections. Each section represents a distinct concept. The concepts are hypothesized to interact and contribute to cancer-related accelerated aging through complex multi-level pathways that may be unidirectional, bidirectional, or moderated. In addition, the relationships between/among the concepts are dynamic, shifting over time and across contexts to influence the aging process. The MMCRAA includes six key concepts: person factors, behavioral factors, biological factors, treatment factors, symptom factors, and life course factors. The evidence to support the association between each of these concepts and variations in accelerated aging in patients with cancer is synthesized and briefly described.

### Assumptions of the MMCRAA

The underlying assumptions of the model are: (1) human development is a lifelong process influenced by biological, psychological, and social factors; (2) the heterogeneity in the health outcomes of oncology patients with the same chronological age reflects differences in their biological/functional age; and (3) the causes and consequences of cancer-related accelerated aging are multifactorial.

### Operational definition of cancer-related accelerated aging

An operational definition describes the procedures and/or outcome(s) that measure a construct^57^. Aging is a universal phenomenon defined as a time-dependent decline in functional capacity and the ability to engage in allostasis or maintain homeostasis^43,59^. Cancer-related accelerated aging refers to a faster rate of decline, such that, compared to the general population, oncology patients exhibit health outcomes associated with aging earlier than expected^60^.

Accelerated aging is operationalized using either clinical measures that estimate functional age (e.g., activities of daily living^61^, Karnofsky Performance Status scale^62^, Functional Assessment of Cancer Therapy–Cognitive)^63^, Deficit Accumulation Index (DAI)^64^, Frailty Index^65^) or biomarkers of aging (e.g., epigenetic clocks^66^, inflammatory markers^67^, proteomic aging clock^68^, neuroimaging^69^) to estimate biological age^23^. Changes in these biological and functional measures correlate with mortality and age-related outcomes in oncology patients and capture inter-individual variability in the underlying biological processes (i.e., hallmarks of aging) associated with aging^70,71^.

## Model components

In the sections that follow, the rationale for the inclusion of each of the concepts in the MMCRAA is briefly summarized. For each of the concepts, specific components within the concepts are illustrated in boxes in Fig. 3.

### Person factors

Oncology patients come to the cancer experience with a unique set of underlying attributes that impact how they respond to their treatment and influence their risk for accelerated aging. Person factors included in the MMCRAA are sex, minoritized status, multimorbidity, psychological traits, and stress.

#### Sex

Evidence suggests that compared to men, women have a longer life expectancy^72,73^. These findings may be related to sex differences in their rate of aging. For example, in a meta-analysis^49^, male sex was associated with increased biologic age estimated using epigenetic clocks trained to predict chronological age (i.e., Hannum, Horvath). However, compared to men, women have a higher prevalence of frailty^74^ and are more likely to experience adverse effects^75^ and a higher symptom burden^76,77^ from their cancer treatment. The resulting male-female health-survival paradox^78^, may be related to sex-chromosomal linked mechanisms^73^, sex-hormonal effects^73^, differences in lifestyles^79^, and/or the biological processes that underlie aging^73,80^. Given the evidence for sex differences in biological aging and health outcomes associated with aging and cancer treatment, it is important that differences in sex are examined when evaluating the relationships between various risk factors and cancer-related accelerated aging.

#### Minoritized status

Minoritized status refers to a patient belonging to a group that holds less social, political, or economic power compared to the dominant or majority group^81^. Minoritized status is related to various characteristics, including race and ethnicity, religion, immigration status, language, sexual orientation, gender identity, disability status, and age. Perceived discrimination based on these characteristics is associated with increased psychosocial stress^82^ and accelerated biological aging. For example, in a study that used growth mixture modeling to characterize distinct subgroups of African American adolescents based on their level of perceived discrimination^83^, membership in the high and stable discrimination group was associated with a higher epigenetic age. Of note, for every one-unit increase in perceived discrimination score, epigenetic age increased by 1.5 years. The authors suggested that repeated experiences of discrimination were likely to trigger the activation of stress response systems.

Studies that evaluated the relationship between discrimination based on minoritized status characteristics and accelerated aging among patients with cancer are limited. In one study that evaluated the relationship between discrimination and the DAI score in African American patients with cancer^84^, as the number of discriminatory events increased, the DAI score increased. These findings suggest that a higher level of perceived discrimination is associated with frailty, a key phenotype of aging.

Given that cancer-related accelerated aging is associated with an earlier onset of functional and cognitive decline and sensory impairment, discrimination based on disability (i.e., ableism) is likely to contribute to increases in both stress and accelerated aging. Evidence to support this hypothesis comes from a national poll that found that adults with disabilities were more likely to report that discrimination was a somewhat or significant source of their stress^82^. Since research that examines the effects of discrimination on biological aging among patients with cancer is limited to one study of African American race^84^, additional studies are needed to validate these findings and evaluate other minoritized characteristics. In addition, studies are needed that evaluate whether multiple intersecting minoritized characteristics contribute to increased psychosocial stress and accelerated aging among patients with cancer.

#### Multimorbidity

In the general population, having a greater number of chronic illnesses is associated with increased accelerated biological aging^85^. For example, for every 7-year increase in the Levine epigenetic clock^85^, the rate of multimorbidity accumulation increased by 6%. In addition, as reported in a meta-analysis^49^, having a higher body mass index, a mental health illness (i.e., depression, schizophrenia), and/or a diagnosis of diabetes, chronic obstructive pulmonary disease, or cardiovascular disease were associated with increased biological age estimated using one or more epigenetic clocks.

While studies demonstrate that a greater number of chronic conditions is associated with increased biological aging in the general population, less is known about these relationships in patients with cancer. In one study that dichotomized patients based on their history of any cancer^48^, a dose-response relationship was observed between increases in the number of co-morbidities and increases in biological aging in both groups, regardless of cancer history. In another study that examined whether biologic age acceleration was associated with an increased risk of developing a chronic health condition over time^27^, compared to patients with cancer in the lowest tertile of biologic age acceleration, patients in the highest tertile had an increased risk of developing hypertension, obesity, myocardial infarction, lung disease, and/or peripheral motor neuropathy. Additional research is needed to determine whether the presence of specific chronic conditions and/or the accumulation of comorbidities as a result of cancer and its treatment increases cancer-related accelerated aging. This research is important because multimorbidity may predispose oncology patients to an accelerated aging trajectory.

#### Psychological characteristics

Psychological characteristics, such as an oncology patient’s level of resilience, perception of well-being, self-perceived health, and personality traits (e.g., conscientiousness, neuroticism), can alleviate or exacerbate stress^86^, inflammatory^87^, and/or multi-omic^88^ mechanisms that contribute to aging. In addition, these moderators influence health behaviors (e.g., smoking, diet) that affect aging^89^.

##### Psychological resilience

Psychological resilience is the ability to adapt to adversity, maintain emotional stability, and thrive in response to difficult or challenging circumstances^90^. In the general population, higher psychological resilience is associated with greater longevity^91^ and slower epigenetic age acceleration^92^. For example, in a study of the general population^86^, higher levels of resilience-related factors (i.e., emotional regulation, self-control) moderated the relationship between higher cumulative stress and increased accelerated biological aging estimated using the GrimAge epigenetic clock. In a study of oncology patients^93^, a decrease in biologic age was significantly correlated with an increase in resilience scores after the completion of a 12-week exercise program. These studies provide some evidence to suggest a relationship between resilience and biological aging. In addition, since lower psychological resilience is associated with higher symptom severity scores and higher cancer-related distress in patients with cancer^94^, resilience may moderate the relationship between symptom severity and distress in patients with cancer and influence cancer-related accelerated aging.

##### Psychological well-being

Psychological well-being encompasses dimensions that describe an individual’s overall level of emotional functioning (e.g., autonomy, environmental mastery, personal growth, purpose in life, and self-acceptance)^95^. In a study of older adults that evaluated the relationships between dimensions of well-being and aging^96^, higher purpose in life was associated with reduced biologic age using four epigenetic clocks (i.e., PhenoAge, GrimAge, Zhang, and Dunedin PoAm). In another study of the general population^97^, among individuals with lower psychological well-being, higher cumulative life stress was associated with increased biological age estimated using the GrimAge2 epigenetic clock. In addition, among individuals with higher psychological well-being, higher levels of cumulative stress did not affect their biological aging. Therefore, because the effect of stress on biological aging may be influenced by perceptions of psychological well-being, these relationships warrant evaluation in patients with cancer.

##### Self-perceived health

Self-perceived health is a patient’s assessment of their overall health and is associated with mortality^98^, psychological well-being^99^, objective measures of physical function^100^, and mechanisms associated with aging (i.e., inflammation)^101^. Because perceived health reflects indicators of health (e.g., physical conditions, emotional states, and symptoms) that are meaningful to an individual^102^, it may reflect aspects of health that are not routinely captured by standard clinical assessments. Discrepancies between oncology patients’ expectations of and perceived declines in health indicators that they deem meaningful may increase stress and negatively impact aging-related outcomes.

A limited body of research evaluated the relationships between self-perceived health and biological aging^30,31,48,103^. For example, in a study of patients with cancer^31^, lower self-reported physical health was associated with accelerated aging estimated using a proteomic aging clock. For every 5-year increase in biological age, self-reported physical health decreased by nearly two points. In another study of the general population^30^, compared to individuals who rated their health as excellent, those who rated their health as poor had a significantly faster pace of biological aging estimated by the DunedinPACE clock. Additional studies are needed to determine whether poor self-perceived health contributes to cancer-related accelerated aging.

##### Personality traits

A large body of research identified specific personality traits associated with poorer health outcomes (i.e., cardiovascular disease, diabetes^104^) and increased mortality risk^105,106^. As noted in a meta-analysis^105^, individuals with scores in the lowest tertile for the conscientiousness personality trait (i.e., low persistence, poor self-control, poor long-term planning) had a 37% increased risk of death compared to those in the top two tertiles. This finding suggests that lower levels of the conscientiousness personality trait may increase the risk for accelerated aging.

Limited evidence suggests that personality traits may influence the aging process by mediating the negative health effects of stress. For example, in a study of the general population^86^, higher cumulative life stress was associated with increased biological age in individuals with higher levels of neuroticism (i.e., the predisposition to experience negative emotions such as anxiety, sadness, worry, and anger). This finding suggests that higher levels of neuroticism contribute to increased vulnerability to the pathologic aging effects of stress. Future studies should evaluate the mediating/moderating effects of personality traits on the relationships between stress and aging in patients with cancer.

### Stress

The relationship between increased levels of psychological stress and the biological mechanisms associated with aging was reviewed extensively^107–111^. Psychological stress refers to the emotional strain or pressure that results from a stressor(s), namely: an experience that an individual perceives as challenging, threatening, or overwhelming. Exposure to a stressor(s) that exceeds an individual’s ability to adapt activates the sympathetic nervous system, inflammatory pathways, and stress axes (e.g., hypothalamic pituitary adrenal axis, sympathetic-adreno-medullary axis) and results in elevated levels of catecholamines, proinflammatory cytokines, and glucocorticoids. These hormones can influence the rate of aging through alterations in epigenetic regulation and chronic inflammation^112^.

A growing body of research demonstrates an association between increased levels of stress and accelerated biological aging in the general population. For example, findings across studies suggest that experiencing stress related to a traumatic event(s) is associated with accelerated biological aging^113–116^. In addition, a diagnosis of post-traumatic stress syndrome is associated with accelerated biological aging^117^. Given that a cancer diagnosis is often a traumatic event in a person’s life and that cancer-related stress can reach the threshold for post-traumatic stress syndrome^118^, an examination of the relationship between stress and accelerated aging in patients with cancer is warranted.

### Behavioral factors

#### Lifestyle

The association between lifestyle behaviors (e.g., poor dietary nutrient intake^71,119,120^, smoking^49,121^, physical inactivity^71^) and accelerated aging is well supported by studies of the general population. However, the evidence linking lifestyle behaviors and accelerated aging in patients with cancer is limited. For example, in one study of oncology patients that evaluated the relationship between five suboptimal health behaviors (i.e., low physical activity, low weight bearing activity, smoking, lack of adherence with a healthy diet, risky drinking) and accelerated aging^27^, compared with cancer survivors with favorable health behaviors, among those with intermediate or unfavorable health behaviors, biologic age was significantly higher. In another study of oncology patients^122^, compared to never smokers, having a history of tobacco use and fewer smoking cessation years was associated with a higher biologic age. Smoking is associated with predictable changes in DNA methylation patterns consistent with accelerated aging^123^. Additional research is needed to validate these findings and investigate interventions aimed at improving lifestyle behaviors and their effect on cancer-related accelerated aging.

#### Coping

Coping strategies are the cognitive, emotional, and/or behavioral efforts that individuals use to respond to and adapt to stress. Coping strategies can be categorized as either engagement or disengagement behaviors. In oncology patients, compared to engagement coping, disengagement coping was associated with a higher symptom burden^124^ and higher levels of stress^125^. While no studies evaluated the relationships between differences in coping behaviors and cancer-related accelerated aging, in a study of African Americans who experienced racial discrimination, compared to individuals who sought social support (i.e., an engagement type coping behavior)^126^, individuals who did not seek out social support were biologically older estimated using an epigenetic clock. In addition, greater use of social support mitigated the effects of discrimination on accelerated aging. Additional research is needed to evaluate the relationships between various types of engagement and disengagement coping behaviors and cancer-related accelerated aging.

### Biological factors

Evidence from animal and human studies identified twelve interrelated biological processes, known as the hallmarks of aging (i.e., genomic instability, telomere attrition, epigenetic alterations, loss of proteostasis, disabled macroautophagy, deregulated nutrient-sensing, mitochondrial dysfunction, cellular senescence, stem cell exhaustion, altered intercellular communication, chronic inflammation, dysbiosis)^43^, that underlie pathological aging. Findings from these studies suggest that when these processes are perturbed or alleviated by experimental interventions^127–129^, they influence the trajectory of aging. Several of the hallmarks of aging are implicated in cancer development and progression^42,130^ and biological markers of their occurrence are observed at disproportionately higher rates in patients receiving cytotoxic treatments^11,131,132^. Therefore, the hallmarks of aging may interact synergistically with the mechanisms that promote tumorigenesis (i.e., hallmarks of cancer^130^) and the pharmacokinetic pathways through which cancer treatments exert their effect(s) to accelerate aging.

The twelve hallmarks of aging and their relationship with cancer were extensively reviewed^133,134^. In the sections that follow, as described previously^43^, each hallmark of aging is categorized as a primary, antagonistic, or integrative factor. Its relationship to accelerated aging is briefly discussed, either in the context of oncology or, when evidence is lacking, in findings from the general population.

#### Primary hallmarks

The primary hallmarks, that include genomic instability, telomere attrition, epigenetic alterations, loss of proteostasis, and loss of macroautophagy, arise from a decline in the efficiency of repair mechanisms and the accumulation of damage caused by various internal and external stressors over time^43^.

##### Genomic instability

Genomic instability refers to a broad range of insults to the structural integrity of genetic material^135^. Genomic instability is a hallmark of both aging^135,136^ and cancer^42,130^. Specifically, DNA damage (e.g., strand breaks, crosslinks) and somatic mutations play distinct roles in aging and cancer. DNA damage represents physicochemical alterations to the DNA molecule. Somatic mutations are stable sequence changes that often arise from replication errors from damaged DNA. While mutations are central to cancer development^137^, persistent DNA damage and transcriptional dysregulation are emerging as primary mechanistic contributors to aging^138,139^.

With advancing age, DNA damage accumulates, resulting in less efficient repair mechanisms, replication errors, and reduced transcriptional output^43,138,139^. This transcriptional suppression effects diverse cellular processes and contributes to multiple hallmarks of aging^138,139^. For example, inherited defects in genomic repair mechanisms are associated with premature aging syndromes (e.g., Werner syndrome^140^, Cockayne syndrome^141^). In addition, mutations arising from errors in the repair process of DNA lesions result in permanent changes to the DNA sequence that contribute to oncogenesis by activating proto-oncogenes, inactivating tumor suppressors, and enabling uncontrolled cell proliferation^42,130^.

In addition, the contribution of DNA damage to aging is reflected in the mechanisms of many cancer treatments. For example, alkylating agents (e.g., cyclophosphamide), commonly used to treat a variety of cancers, inhibit cell division by inducing cross-links between guanine bases on DNA strands^142^. Another class of drugs, poly ADP-ribose polymerase inhibitors^143^, block enzymes involved in DNA repair processes.

##### Telomere attrition

Telomeres, protective caps at the end of chromosomes, progressively shorten with each cell division^144^. Upon reaching a critically reduced length, telomeres trigger cellular responses that lead to apoptosis or cell cycle arrest^144^. Genetic defects that result in the reduction of telomerase, an enzyme that maintains the structural integrity of telomeres, are associated with cancer and degenerative diseases (e.g., Dyskeratosis congenita)^145^.

As noted in one review^146^, the relationships between cancer treatments and changes in telomere length are inconsistent. The authors concluded that the reasons for these inconsistent findings may be related to differences in cancer types and treatment(s). For example, mitotic inhibitors (e.g., taxanes^147^) may cause telomere deprotection (i.e., uncapping) while hematopoietic growth factors (e.g., granulocyte colony-stimulating factor^148^) may upregulate telomerase and result in telomere elongation. In addition, the upregulation of telomerase is one way that cancer cells evade mortality^42^, and the amount of telomerase upregulation varies by cancer type. For example, telomerase reverse transcriptase gene promoter mutations are most common among patients with bladder cancer and least common among patients with colorectal cancer^149^. Equally important, because changes in telomere length are associated with lifestyle factors (e.g., exercise, diet) and stress^150^, these factors may contribute to variations in telomere length among oncology patients. While findings regarding the effects of cancer treatment on telomere length are inconclusive, telomere attrition appears to be a risk factor for accelerated aging^151^.

##### Epigenetic modifications

Epigenetic modifications (i.e., DNA methylation, histone modification, chromatin remodeling, non-coding ribonucleic acid (i.e., RNA) dysfunction) contribute to genomic instability, and these alterations are observed in both aging^43^ and cancer^130^. For example, increasing age is associated with global hypomethylation, loss of heterochromatin, and changes in histone acetylation and microRNA expression^43^. These epigenetic alterations contribute to the transcriptional silencing of genes involved in DNA repair and maintenance, as well as the upregulation of genes involved in proinflammatory pathways^43^. In addition, variations in the epigenome are associated with external factors (e.g., toxins, diet, exercise, substance use, and stress)^152^.

While evidence from epigenome-wide association studies suggests predictable patterns of epigenetic modification across the lifespan^43,152^, changes in DNA methylation patterns are the most widely studied and predictive of chronologic age^60,153^, mortality^154^, and aging phenotypes^71^. Epigenetic clocks estimate biological age using DNA methylation patterns^66^. These estimates are correlated with cancer risk and survival^155^, mortality^156^, and diseases associated with aging^157,158^. In addition, studies of oncology patients demonstrate that epigenetic age increases from pre- to post-treatment^25,122,159,160^.

##### Loss of proteostasis and macroautophagy

Loss of proteostasis (i.e., the mechanisms by which proteins are stabilized or degraded)^161^ and loss of macroautophagy (i.e., the processes by which cells break down and recycle damaged cellular material)^162^ are associated with advancing chronological age^162^ and neurodegenerative conditions associated with aging (e.g., Alzheimer’s disease^162^). In addition, cancer cells^163^ and cancer treatments^164,165^ disrupt protein folding homeostasis by inducing genomic instability and/or disabling macroautophagy. Loss of these critical processes leads to the accumulation of damaged macromolecules (e.g., misfolded proteins, dysfunctional organelles) and contributes to chronic inflammation^161^. Proteomic aging, estimated using patterns of plasma protein expression to predict biological age^166^, is associated with increased risk for multimorbidity, frailty, and mortality in the general population^68^. In addition, in a study of oncology patients^166^, a one standard deviation increase in proteomic aging was associated with a 56% increased risk of all-cause mortality.

#### Antagonistic hallmarks

The antagonistic hallmarks are the systemic consequences of the primary hallmarks and include cellular senescence, mitochondrial dysfunction, and deregulated nutrient-sensing.

##### Cellular senescence

Cellular senescence is a permanent state of cell cycle arrest that can be triggered by the primary factors^43^. Senescent cells remain metabolically active and secrete proinflammatory cytokines, chemokines, extracellular matrix fragments, and degrading enzymes, described as the senescence-associated secretory phenotype (i.e., SASP)^167^. SASPs can induce senescence in surrounding cells^168^. A large body of research demonstrates that the p16 cyclin-dependent kinase inhibitor 2 A protein (i.e., p16 INK4a), a biological marker of cellular senescence, increases with increasing chronological age^169^ and after cancer treatment (e.g., stem cell transplant^170,171^, chemotherapy^170,172,173^). Across these studies, the difference in p16 INK4a expression from pre- to post-treatment levels represented a 14^173^ to 35^172^ year age acceleration.

##### Deregulated nutrient sensing

Alterations in metabolism, as a result of deregulated nutrient sensing, are implicated in aging^174^ and cancer progression^175,176^. The nutrient-sensing network responds to the availability of nutrients through complex cellular signaling pathways to mediate metabolism (i.e., anabolism, catabolism). Deregulated nutrient sensing is associated with increased epigenetic age in human cells^177^. In addition, pharmaceuticals (e.g., rapamycin, metformin) that inhibit key nutrient-sensing pathways (e.g., Insulin-like Growth Factor 1, mechanistic Target of Rapamycin Complex 1) are associated with a longer lifespan in animal models^174^. In human studies^174^, centenarians were more likely to carry loss-of-function genetic variants in Insulin-like Growth Factor/Insulin signaling nutrient-sensing pathways.

##### Mitochondrial dysfunction

Similarly, mitochondrial dysfunction is associated with metabolic alterations (e.g., impaired energy production). Dysfunctional mitochondria can result from oxidative stress^178^ and/or the accumulation of mutations in mitochondrial DNA^179^. Damaged mitochondria release increased levels of reactive oxygen species (i.e., ROS)^178^. This increase in the production of ROS disrupts redox homeostasis, resulting in cellular dysfunction and/or cell cycle arrest (i.e., senescence)^180^. Cancer cells produce high levels of ROS due to their rapid proliferation^181^. Many chemotherapeutics (e.g., doxorubicin^182^ cisplatin^183^) exert their anti-neoplastic effects by increasing oxidative stress beyond the cancer cells’ ability to function. In a mouse model^184^, compared to controls, doxorubicin-induced cardiotoxicity was associated with a significant increase in mitochondrial damage and markers of senescence immediately after treatment. In addition, these increased markers of senescence persisted after treatment. Furthermore, a study that compared cardiac function in patients who were and were not exposed to doxorubicin found that exposed patients had changes in cardiac function comparable to older adults^185^.

#### Integrative hallmarks

The integrative hallmarks (i.e., chronic inflammation, dysbiosis, stem cell exhaustion, altered intercellular communication) refer to the cumulative effects of the primary and antagonistic hallmarks^43^. For example, senescence can be triggered by many of the primary hallmarks^167,186^ and results in a chronic inflammatory state (i.e., inflammaging)^187^. Elevated levels of inflammatory markers (e.g., C-reactive protein, interleukin-6) were observed in oncology patients undergoing radiation^25^ and chemotherapy treatment^188^. These increases were associated with increases in accelerated biological aging^25,188^.

##### Dysbiosis

Chronic inflammation is further exacerbated by an imbalance or disruption in the composition of the intestinal microbiota (i.e., dysbiosis)^189^, which can be a risk factor for cancer^190^ and can occur as a result of aging^191^ and/or cancer treatment(s)^192^. Cancer treatments can damage the intestinal mucosa, making it more permeable to toxic substances. While direct evidence linking dysbiosis to cancer-related accelerated aging is not available, changes in the composition of intestinal microbiota are associated with frailty^193^, cognitive decline^194,195^, and the hallmarks of aging (i.e., altered nutrient sensing, cellular senescence) in the general population^43^. In addition, emerging evidence suggests that distinct microbiome signatures are correlated with increased accelerated biological aging in the general population^196,197^ and in patients with Human Immunodeficiency Virus^198^.

##### Stem cell exhaustion

Stem cells are essential for tissue renewal and repair. Aging, chronic inflammation, and genomic instability are associated with a decline in the number and function of stem cells^43^. In contrast, proliferative signaling by oncogenes is associated with stem cell maintenance and tumorigenesis^42^. Cancer treatments contribute to the depletion or dysfunction of hematopoietic stem cells. For example, in a study of patients with hematologic malignancies treated with Cluster of Differentiation 19 Chimeric Antigen Receptor T (i.e., CD19 CAR-T) cells^199^, 16% of patients experienced persistent cytopenias that lasted for at least 1 year after treatment.

##### Altered cellular communication

Alterations in cellular communication are a consequence of both the hallmarks of aging^43^ and cancer and its treatment(s)^42^. For example, the accumulation of senescent cells and misfolded proteins that occur with increasing age is known to contribute to dysregulated paracrine and autocrine signaling^168^. In addition, cancer cells may evade immune surveillance by altering receptors involved in immune recognition. Emerging immunotherapies (e.g., immune checkpoint inhibitors, CAR T-cell therapy) exploit these alterations to promote active immunity against cancer cells. While effective, immunotherapies are associated with overactivation of the immune system, chronic inflammation, and hematologic toxicity^200^, which may contribute the cancer-related accelerated aging.

### Treatment factors

As discussed in the previous section, cancer treatments impact multiple hallmarks of aging through diverse mechanisms. Several factors, such as the type(s), number of treatments (i.e., combination, recurrent), and intensity (i.e., duration, number of cycles, length of time between cycles/treatments) of the treatment regimen(s), contribute to cancer-related accelerated aging.

#### Type(s)

The aging effects of cancer treatments largely depend on their mechanism(s) of action and adverse effects. Chemotherapy and radiation, due to their nonspecific genotoxic and cytotoxic effects, disrupt nearly all of the hallmarks of aging. These therapies contribute to the accumulation of replicative senescence that results in a chronic inflammatory state (i.e., inflammaging). Similarly, surgical interventions trigger the activation of the inflammatory cascade^123^ and are associated with increases in SAPS^201^. Hormone therapies (e.g., tamoxifen, aromatase inhibitors) may modulate nutrient-sensing networks^202^. Targeted therapies (e.g., kinase inhibitors, monoclonal antibodies) and immunotherapies (e.g., immune checkpoint inhibitors) alter proteostasis, epigenetic regulation, and intracellular signaling networks, which in turn can accelerate aging^203^.

#### Number of treatment(s)

Many oncology patients receive multiple treatments either in combination, succession, or to treat secondary cancers during the course of their illness. In several studies, the receipt of more than one type of cancer treatment was associated with an increased incidence of severe toxicity^204^, poorer health outcomes (i.e., multimorbidity^205^), and increases in accelerated aging^206^.

#### Intensity

Most cancer treatments are administered in successive cycles (e.g., chemotherapy) or fractions (e.g., radiation therapy), which consist of a treatment period followed by a rest period. Increased dose intensity (e.g., higher dose, shorter time between rest periods, longer duration of treatment cycles) contributes to an increase in the toxicity of the treatment regimen. For example, cardiotoxicity following anthracycline treatment demonstrates a dose-dependent, stepwise increase in incidence with increasing cumulative exposure^207^. Increased treatment toxicity is hypothesized to increase the risk of cancer-related accelerated aging^131,208^.

### Symptom factors

On average, oncology patients report 10^209^ to 15^210^ unrelieved symptoms associated with their cancer and its treatment. Higher symptom burden in oncology patients is associated with increased stress^94^, multimorbidity^211^, functional decline^212^, and decrements in quality of life^213^. Commonly assessed dimensions of the symptom experience include patient-reported levels of severity and distress^214^. Findings from a limited number of studies of patients with cancer report that higher levels of fatigue^25,160^, pain^160^, sleep disturbance^160^, peripheral motor neuropathy^27^, cognitive impairment^160^, depression^160^, and anxiety^160^ were associated with increased biologic aging estimated using epigenetic clocks. In addition, in oncology patients, higher levels of common cancer-related symptoms (e.g., fatigue) were associated with the occurrence of clinical frailty^215^, increased levels of inflammatory markers^25^, and variations in microbiome composition (i.e., dysbiosis)^216^. Symptoms may interact with aging-related biological mechanisms in a way that amplifies or modifies their impact on pathologic aging in patients with cancer. More research is needed to understand how cancer-related symptoms, including how severe or distressing they are, are linked to faster aging caused by cancer.

Importantly, symptoms in patients with cancer often co-occur in clusters^217^. Multiple co-occurring symptoms are associated with increased risk for mortality in patients with cancer^218^. In one study of oncology patients^122^, a higher severity of a head and neck treatment-related symptom cluster was associated with increased biologic aging. Given the paucity of research on associations between symptoms, symptom clusters, and cancer-related age acceleration, additional studies are needed.

In addition to symptoms related to cancer and its treatment, a positive relationship was found between post-traumatic stress symptoms (e.g., intrusive thoughts, avoidance, hypervigilance) and increases in biologic aging in non-cancer cohorts^117,219^. Since a higher symptom burden in patients with cancer is associated with an increased prevalence of post-traumatic stress symptoms, (LM, SW, CR, MW, Cooper, B., Hammer, M., Conley, Y., Steven, P., Levine, J., CM, in review) an evaluation of the relationship between post-traumatic stress symptoms and accelerated biological aging is warranted in patients with cancer.

### Life course factors

**Fig. 4:**
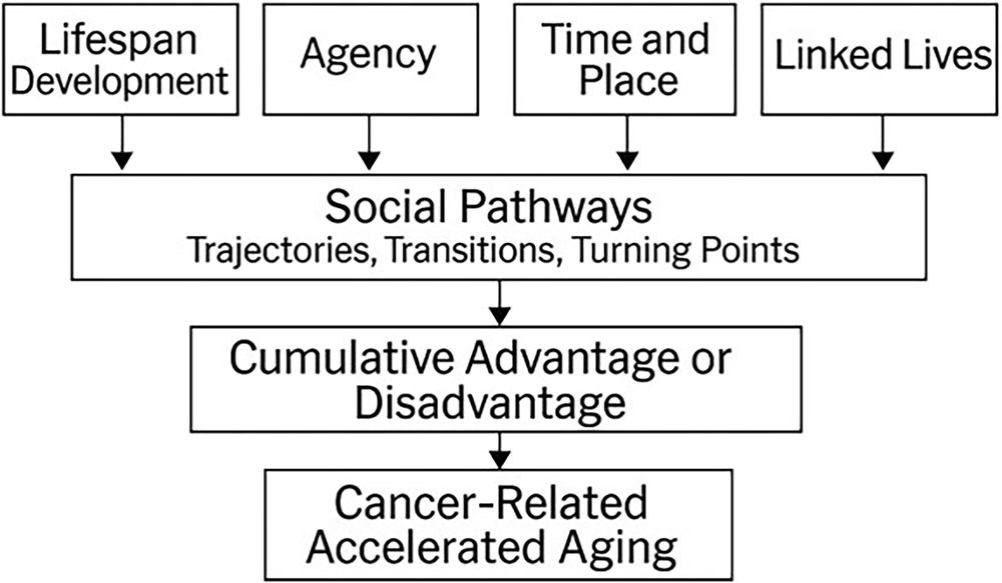
Application of the Life Course Theory to the Multifactorial Model of Cancer-Related Accelerated Aging.

The Life Course Theory is a multidisciplinary framework that describes how various factors, including individual characteristics, social structures, environmental influences, and historic contexts, interact to shape people’s lives over time^45^. The Life Course Theory evolved from longitudinal studies of childhood development that examined the relationships between experiencing a disruptive societal event (e.g., war, economic downturn) and health and socioeconomic outcomes^45^. These studies revealed that health and aging are influenced by social pathways and several core principles (i.e., lifespan development, human agency, time and place, linked lives). Differences in social pathways contribute to the accumulation of advantages and/or disadvantages over time. As shown in Fig. 4, the Life Course Theory can be applied to oncology research to describe the heterogeneity in aging trajectories observed among patients with cancer.

#### Social pathways

A social pathway refers to the trajectories and transitions of roles that are followed by groups of people across time. Successful management of life transitions is associated with greater psychological well-being^220^. Social pathways are shaped by social institutions and cultural norms, and expectations. A turning point, such as a cancer diagnosis, is a significant and often stressful life event that contributes to transitions in an oncology patient’s role (e.g., occupational, social, and family). Other significant turning points in oncology patients’ lives are adverse childhood experiences and stressful life events. As noted in several reviews^50,52,221,222^, adverse social exposures across the lifespan result in accelerated aging in the general population. Additional research is warranted that examines the relationships between stressful life events and cancer-related stress on cancer-related accelerated aging.

#### Lifespan development

The principle of lifespan development emphasizes that people continue to grow, change, and adapt throughout their lives. Each stage of life builds upon prior experiences and impacts future developments. While age-related patterns exist, people can change at any point in life, especially in response to a significant event. For example, transitions, such as becoming a patient and dealing with a cancer diagnosis, affect later stages of life. In addition, some patients may experience poor role functioning as a result of their cancer diagnosis and its treatment. Among oncology patients, poor role functioning (i.e., the inability to fulfill their expected roles in various aspects of life) is associated with increased stress^223^, increased mortality^224^, and decrements in quality of life^223^ and may accelerate aging in oncology patients. Future research is needed that examines the relationship between oncology patients’ role functioning and cancer-related accelerated aging.

#### Agency

The principle of agency recognizes that, within the opportunities and constraints of their circumstances, individuals make decisions and exert control over their life course trajectory. Agency in oncology patients is exercised through their decision to adhere to or deviate from treatment plans^225^ and/or health-promoting behaviors^226^. In addition, oncology patients may utilize either engagement or disengagement coping strategies to respond and adapt to their cancer diagnosis and treatment^124^. While patients act independently, their decisions, values, and preferences are influenced by their environment and social context^226^. For example, the developmental psychology literature suggests that when individuals are faced with a chronic or uncontrollable stressor(s) (e.g., poverty, violence, and family conflict), they are more likely to use disengagement type coping strategies (e.g., substance use, avoidance, and distraction) because these strategies provide immediate relief from the stressor and/or allow for adaptive functioning within challenging environments^227,228^. While important for survival in certain contexts, disengagement coping is associated with poorer health outcomes^229^ and engagement coping strategies (e.g., seeking support^126^) are protective against accelerated aging.

#### Time and place

The principle of time and place refers to shifts in the meaning of a life event (i.e., transition, turning point) depending on the developmental stage and social geographic context of the individual^45^. For example, compared to older adults, younger adults experience higher levels of distress as a result of their cancer diagnosis and/or treatment(s)^118,230,231^ and shifts in their role functioning^231^. These differences may be related to changes in emotional regulation^231^, coping^232^, life roles^233^, and psychological resilience^234,235^ across the different developmental stages of life. In terms of self-perceived health, indicators of health that patients deem as meaningful may change over time^236^ and are shaped by previous health experiences^237^ and cultural factors^238^. In addition, evidence suggests that levels of resilience^234^ and coping behaviors^239^ change with age. Therefore, psychological characteristics and behavioral factors may be influenced by the principle of time and place and are hypothesized to contribute to increased cancer-related accelerated aging.

Another aspect of the principle of time is the concept of a critical or sensitive period through which the effects of an event or exposure have an increased impact on a health outcome^240^. In oncology patients, evidence suggests that the rate of accelerated aging varies depending on the chronological age of the patient at the time of their cancer diagnosis. For example, compared to healthy controls^241^, survivors of childhood cancer were estimated to be between 5.2 and 15.9 years older using epigenetic clocks. In another study^242^, compared to healthy controls, older breast cancer survivors were estimated to be between 1.04 and 2.22 years older using epigenetic clocks. Additional research is needed to understand the impact of the timing of the cancer diagnosis, as well as various stressors on accelerated aging trajectories.

Within the principles of time and place, place refers to a patient’s physical, social, and cultural environment. Past injustices experienced by minoritized communities (e.g., discrimination, intergenerational cultural trauma^243^) can affect how patients perceive and respond to their care. Cultural beliefs and values that conflict with the views of the dominant healthcare system can increase psychosocial stress and contribute to poorer health outcomes^244^. In addition, neighborhood characteristics (e.g., social cohesion, exposure to crime) contribute to levels of stress and aging trajectories^245,246^. Additional research is needed to explore the relationships between the principle of time and place and cancer-related accelerated aging.

#### Linked lives

The principle of linked lives highlights how life course trajectories are interdependent on others within their sociocultural network. For example, patients may rely on the help of an informal (i.e., unpaid) caregiver to assist with their health care needs or to provide emotional support. As noted in two reviews^247,248^, when the responsibilities of caregiving surpass their capacity to cope, being an informal caregiver is associated with poor health outcomes (i.e., depression, multimorbidity, frailty). Importantly, a bidirectional relationship exists between the emotional and physical well-being of informal caregivers and the health outcomes of patients with cancer^249^. For example, oncology patients, whose informal caregiver reported higher levels of depressive symptoms or poorer self-rated health, had an increased odds (i.e., 1.06 and 3.76, respectively) of reporting poorer quality of life^250^. Given that a lower quality of life among oncology patients is associated with a higher risk for mortality^251^ and accelerated aging^122^, the relationship between the patient and informal caregiver is an important focus of future research so that interventions can be developed to support the needs of both of them.

#### Cumulative advantages and disadvantages

It is well documented that life expectancy and health outcomes vary widely across demographic groups^252^. These disparities are shaped by the conditions into which people are born, live, work and age as well as their access to resources. In addition, differences in how patients adapt to life transitions and turning points, and the timing of these events with respect to their stage of development, contribute to the accumulation of advantages and/or disadvantages over time. For example, having a lower socioeconomic status^49^, lower income^48^, lower educational attainment^48,49,222^, higher neighborhood deprivation^221^, and/or increased exposure to air pollution^49^ were associated with accelerated aging. In a study of oncology patients^48^, those individuals with unmet health-related social needs (e.g., higher food insecurity, lack of insurance coverage) were accelerated in their age estimated using the PhenoAge epigenetic clock.

Life Course Theory posits that these exposures, disparities, and/or experiences become “embodied” or “inscribed” in the physiological processes that influence aging through “chains of risk” ^240^. Chains of risk models describe how an initial event increases the likelihood of a subsequent event(s) and how these accumulated events can either compound disease risk over time or exert independent effects on health^240^.

## Future directions for clinical practice and research

The identification of risk factors associated with cancer-related accelerated aging has important implications for clinical practice. The ability to identify patients who are most vulnerable to increased cancer-related accelerated aging can help guide treatment decisions, facilitate discussions about the effects of treatments on life expectancy and quality of life, and inform supportive care strategies. Oncology patients and clinicians can use this information to develop personalized treatment plans that align with the patients’ priorities, such as preserving functional status or extending the healthspan.

As summarized in Table 1, several recommendations for future research should be prioritized. Future research is needed to characterize the cancer-related accelerated aging phenotype using a life course perspective. Importantly, a need exists to develop a consensus on a standardized, valid, and feasible measure of accelerated aging in patients with cancer that can be used in clinical and research settings. In addition, a need exists to create a comprehensive, multidimensional index of cancer-related accelerated aging that integrates person, behavioral, biological, treatment, symptom, and life course factors. Determining the relative contribution of each factor in the MMCRAA will improve the predictive accuracy of tools used to estimate biological aging in oncology populations. Future work must define clinically meaningful changes in accelerated aging and determine how these changes relate to outcomes such as symptom burden, functional decline, and quality of life.

**Table 1:** Recommendations for future research on cancer-related accelerated aging

Research Topic	Recommendation
Conceptual	■ Determine the relative contribution of each of the MMCRAA factors in predicting cancer-related accelerated aging. ■ Develop a multidimensional composite index of cancer-related accelerated aging that includes a broad range of predictors (i.e., person factors, behavioral factors, biological factors, treatment factors, symptom factors, and life course factors) and validate this index using biologic and functional estimates of aging.
Methodological	■ Determine the clinically meaningful change associated with increases in cancer-related accelerated aging and differences in symptom burden and quality of life. ■ Achieve consensus among clinicians and researchers on which clinical and biological measures are most feasible, reliable, and valid to use to estimate accelerated aging in patients with cancer.
Person factors	
Sex	■ Evaluate sex differences in cancer-related accelerated aging. ■ Evaluate for differences in underlying sex-linked mechanisms (e.g., sex-chromosomal linked, hormonal) that may contribute to sex differences in cancer-related accelerated aging.
Minoritized status	■ Evaluate the relationship between discrimination based on diverse minoritized characteristics and cancer-related accelerated aging. ■ Determine whether multiple intersecting minoritized characteristics increase stress and impact cancer-related accelerated aging.
Multimorbidity	■ Evaluate the impact of the various types of chronic conditions and the number of co-morbidities on cancer-related accelerated aging.
Psychological characteristics	■ Evaluate the role of psychological characteristics (i.e., resilience, well-being, self-perceived health status, personality traits) in mediating/moderating the relationship between cancer-related accelerated aging and other risk factors (e.g., symptoms, stress). ■ Investigate the associations between differences in psychological traits (i.e., resilience, well-being, self-perceived health status, personality traits) and the hallmarks of aging. ■ Determine whether interventions aimed at enhancing resilience, well-being, and/or self-perceived health status alleviated increases in cancer-related accelerated aging.
Stress	■ Investigate the relationships among cumulative life stress, traumatic stress, cancer-related stress, and cancer-related accelerated aging.
Behavioral factors	
Lifestyle	■ Evaluate the associations between lifestyle behaviors (e.g., diet, activity) and cancer-related accelerated aging. ■ Evaluate the effect of interventions aimed at improving lifestyle behaviors on cancer-related accelerated aging.
Coping	■ Evaluate the relationships between various types of engagement and disengagement coping behaviors and cancer-related accelerated aging. ■ Determine whether enhancing the use of engagement type coping behaviors is related to decreases in cancer-related accelerated aging.
Biological factors	■ Investigate the associations between the hallmarks of aging and MMCRAA factors. ■ Determine whether primary, antagonist, or integrative biological factors have a stronger effect on accelerated aging.
Treatment factors	■ Investigate the relationships between various types of treatment/combination treatments and their impact on cancer-related accelerated aging. ■ Evaluate the relationships between treatment intensity and cancer-related accelerated aging.
Symptom factors	■ Investigate the relationships between symptoms (i.e., cancer-related symptoms, post-traumatic stress symptoms, symptom clusters) and cancer-related accelerated aging. ■ Evaluate the impact of different dimensions of the symptom experience (i.e., severity, distress) on cancer-related accelerated aging.
Life course factors	■ Incorporate variables that measure the principles of human agency and time and place variables in studies that evaluate the relationships between behavioral factors (diet, activity, coping) and psychological traits (resilience, wellbeing, self-perceived health) and cancer-related accelerated aging. ■ Incorporate variables that measure the principles of time and place to evaluate the relationships between discrimination based on minoritized status and cancer-related accelerated aging. ■ Incorporate variables that measure the principles of time and place to evaluate the relationships between the timing of the cancer diagnosis and/or life stressors on accelerated aging trajectories. ■ Incorporate variables that measure the principles of linked lives to evaluate the relationships between informal caregivers’ characteristics (e.g., stress, coping) and oncology patients’ cancer-related accelerated aging.

## Conclusion

The MMCRAA provides a holistic framework that can be used by clinicians and researchers to improve personalized cancer care and reduce the deleterious effects of cancer and its treatment on accelerated aging and quality of life. By conceptualizing health as a multidimensional phenomenon, future studies can more accurately identify deviations from expected aging processes in patients with cancer. Advances in characterizing and estimating cancer-related accelerated aging will enable the development of targeted interventions. Incorporating life course principles, such as human agency, time and place, and linked lives, will provide an increased understanding of how social context and timing shape aging trajectories in patients with cancer.

## Data Availability

All data supporting this qualitative synthesis are contained within the articles and supplementary materials cited in the manuscript.

## References

[1] Santucci, C. et al. Progress in cancer mortality, incidence, and survival: a global overview. *Eur. J. Cancer Prev.* **29**, 367–381 (2020). 32740162 10.1097/CEJ.0000000000000594

[2] Bhakta, N. et al. The cumulative burden of surviving childhood cancer: an initial report from the St Jude Lifetime Cohort Study (SJLIFE). *Lancet* **390**, 2569–2582 (2017). 28890157 10.1016/S0140-6736(17)31610-0 PMC5798235

[3] van der Meer, D. J. et al. Patient-reported outcomes of accelerated aging: a novel approach to investigate second cancer risk in adolescent and young adult (18-39 Years) cancer survivors. *Oncologist* **29**, e526–e534 (2024). 37995309 10.1093/oncolo/oyad307 PMC10994290

[4] Garmany, A. & Terzic, A. Global healthspan-lifespan gaps among 183 world health organization member states. *JAMA Netw. Open* **7**, e2450241–e2450241 (2024). 39661386 10.1001/jamanetworkopen.2024.50241 PMC11635540

[5] Plasencia, G., Gray, S. C., Hall, I. J. & Smith, J. L. Multimorbidity clusters in adults 50 years or older with and without a history of cancer: national health Interview Survey, 2018. *BMC Geriatr.* **24**, 50 (2024). 38212690 10.1186/s12877-023-04603-9 PMC10785430

[6] Ahmad, T. A., Gopal, D. P., Chelala, C., Dayem Ullah, A. Z. & Taylor, S. J. Multimorbidity in people living with and beyond cancer: a scoping review. *Am. J. Cancer Res.* **13**, 4346–4365 (2023). 37818046 PMC10560952

[7] Bluethmann, S. M., Mariotto, A. B. & Rowland, J. H. Anticipating the “Silver Tsunami”: Prevalence trajectories and co-morbidity burden among older cancer survivors in the United States. *Cancer Epidemiol. Biomark. Prev.* **25**, 1029–1036 (2016). 10.1158/1055-9965.EPI-16-0133 PMC4933329 27371756

[8] Ness, K. K. et al. Physiologic frailty as a sign of accelerated aging among adult survivors of childhood cancer: a report from the St Jude Lifetime cohort study. *J. Clin. Oncol.* **31**, 4496–4503 (2013). 24248696 10.1200/JCO.2013.52.2268 PMC3871511

[9] Kerstens, C. et al. A systematic review on the potential acceleration of neurocognitive aging in older cancer survivors. *Cancers* **15**, 10.3390/cancers15041215 (2023). 10.3390/cancers15041215 PMC9954467 36831557

[10] Siddique, A., Simonsick, E. M. & Gallicchio, L. Functional decline among older cancer survivors in the Baltimore longitudinal study of aging. *J. Am. Geriatr. Soc.* **69**, 3124–3133 (2021). 34346072 10.1111/jgs.17369 PMC8595548

[11] Ness, K. K. et al. Premature physiologic aging as a paradigm for understanding increased risk of adverse health across the lifespan of survivors of childhood cancer. *J. Clin. Oncol.* **36**, 2206 (2018). 29874132 10.1200/JCO.2017.76.7467 PMC6553838

[12] Sedrak, M. S., Kirkland, J. L., Tchkonia, T. & Kuchel, G. A. Accelerated aging in older cancer survivors. *J. Am. Geriatr. Soc.* **69**, 3077–3080 (2021). 34534355 10.1111/jgs.17461 PMC8595814

[13] Muhandiramge, J., Orchard, S., Haydon, A. & Zalcberg, J. The acceleration of ageing in older patients with cancer. *J. Geriatr. Oncol.* **12**, 343–351 (2021). 32933870 10.1016/j.jgo.2020.09.010

[14] López-Otín, C., Blasco, M. A., Partridge, L., Serrano, M. & Kroemer, G. The hallmarks of aging. *Cell* **153**, 1194–1217 (2013). 23746838 10.1016/j.cell.2013.05.039 PMC3836174

[15] Jylhävä, J., Pedersen, N. L. & Hägg, S. Biological age predictors. *EBioMedicine* **21**, 29–36 (2017). 28396265 10.1016/j.ebiom.2017.03.046 PMC5514388

[16] Fries, J. F. in *Handboo*k of *th*e Bio*logy of Aging* (eds Masoro, E. J. & Austad, S. N.) 507–524 (Elsevier Inc, 2016).

[17] Kocarnik, J. M. et al. Cancer Incidence, mortality, years of life lost, years lived with disability, and disability-adjusted life years for 29 cancer groups from 2010 to 2019: a systematic analysis for the Global Burden of disease study 2019. *JAMA Oncol.* **8**, 420–444 (2022). 34967848 10.1001/jamaoncol.2021.6987 PMC8719276

[18] Force, L. M. et al. The global burden of childhood and adolescent cancer in 2017: an analysis of the Global Burden of Disease Study 2017. *Lancet Oncol.* **20**, 1211–1225 (2019). 31371206 10.1016/S1470-2045(19)30339-0 PMC6722045

[19] Hung, M.-C., Lai, W.-W., Chen, H. H. W., Su, W.-C. & Wang, J.-D. Comparison of expected health impacts for major cancers: integration of incidence rate and loss of quality-adjusted life expectancy. *Cancer Epidemiol.* **39**, 126–132 (2015). 25553846 10.1016/j.canep.2014.12.004

[20] Tonorezos, E. et al. Prevalence of cancer survivors in the United States. *JNCI J. Natl. Cancer Inst.* **116**, 1784–1790 (2024). 39002121 10.1093/jnci/djae135 PMC11542986

[21] Cesari, M. et al. Early detection of accelerated aging and cellular decline (AACD): a consensus statement. *Exp. Gerontol.* **146**, 111242 (2021). 33484892 10.1016/j.exger.2021.111242

[22] Guida, J. L. et al. Strategies to prevent or remediate cancer and treatment-related aging. *J. Natl. Cancer Inst.* **113**, 112–122 (2021). 32348501 10.1093/jnci/djaa060 PMC7850536

[23] Guida, J. L. et al. Measuring aging and identifying aging phenotypes in cancer survivors. *J. Natl. Cancer Inst.* **111**, 1245–1254 (2019). 31321426 10.1093/jnci/djz136 PMC7962788

[24] Ness, K. K. & Wogksch, M. D. Frailty and aging in cancer survivors. *Transl. Res.* **221**, 65–82 (2020). 32360946 10.1016/j.trsl.2020.03.013 PMC7321876

[25] Xiao, C. et al. Epigenetic age acceleration, fatigue, and inflammation in patients undergoing radiation therapy for head and neck cancer: a longitudinal study. *Cancer* **127**, 3361–3371 (2021). 34027995 10.1002/cncr.33641

[26] Yang, G. S. et al. Exploring the relationship between DNA methylation age measures and psychoneurological symptoms in women with early-stage breast cancer. *Support. Care in Cancer* **31**, 10.1007/s00520-022-07519-z (2023). 10.1007/s00520-022-07519-z 36538110

[27] Qin, N. et al. Epigenetic age acceleration and chronic health conditions among adult survivors of childhood cancer. *JNCI J. Natl. Cancer Inst.* **113**, 597–605 (2021). 32970815 10.1093/jnci/djaa147 PMC8096366

[28] Montross, L. P. et al. Correlates of self-rated successful aging among community-dwelling older adults. *Am. J. Geriatr. Psychiatry* **14**, 43–51 (2006). 16407581 10.1097/01.JGP.0000192489.43179.31

[29] Strawbridge, W. J., Wallhagen, M. I. & Cohen, R. D. Successful aging and well-being: self-rated compared with Rowe and Kahn. *Gerontologist* **42**, 727–733 (2002). 12451153 10.1093/geront/42.6.727

[30] Belsky, D. W. et al. DunedinPACE, a DNA methylation biomarker of the pace of aging. *Elife* **11**, e73420 (2022). 35029144 10.7554/eLife.73420 PMC8853656

[31] Solomon, M. et al. Cross-sectional associations of proteomic age acceleration with self-reported physical and mental health and depression symptoms among those with and without cancer. *J. Cancer Surviv.*10.1007/s11764-025-01803-7 (2025). 10.1007/s11764-025-01803-7 PMC13676640 40304866

[32] Lu, W., Pikhart, H. & Sacker, A. Domains and measurements of healthy aging in epidemiological studies: a review. *Gerontologist* **59**, e294–e310 (2019). 29897451 10.1093/geront/gny029 PMC6630160

[33] Kanungo, M. S. A model for ageing. *J. Theor. Biol.* **53**, 253–261 (1975). 1195761 10.1016/s0022-5193(75)80002-6

[34] Hayflick, L. & Moorhead, P. S. The serial cultivation of human diploid cell strains. *Exp. Cell Res.* **25**, 585–621 (1961). 13905658 10.1016/0014-4827(61)90192-6

[35] Olovnikov, A. M. A theory of marginotomy: the incomplete copying of template margin in enzymic synthesis of polynucleotides and biological significance of the phenomenon. *J. Theor. Biol.* **41**, 181–190 (1973). 4754905 10.1016/0022-5193(73)90198-7

[36] Harley, C. B., Futcher, A. B. & Greider, C. W. Telomeres shorten during ageing of human fibroblasts. *Nature* **345**, 458–460 (1990). 2342578 10.1038/345458a0

[37] Medawar, P. B. *An unsolved problem of biology*. (H K Lewis & CO, 1952).

[38] Eigen, M. Selforganization of matter and the evolution of biological macromolecules. *Naturwissenschaften* **58**, 465–523 (1971). 4942363 10.1007/BF00623322

[39] Kirkwood, T. B. Evolution of ageing. *Nature* **270**, 301–304 (1977). 593350 10.1038/270301a0

[40] Harman, D. Aging: a theory based on free radical and radiation chemistry. *Sci. Aging Knowl. Environ.* **2002**, cp14–cp14 (2002). 10.1093/geronj/11.3.298 13332224

[41] Cupit-Link, M. C. et al. Biology of premature ageing in survivors of cancer. *ESMO open* **2**, e000250 (2017). 29326844 10.1136/esmoopen-2017-000250 PMC5757468

[42] López-Otín, C., Pietrocola, F., Roiz-Valle, D., Galluzzi, L. & Kroemer, G. Meta-hallmarks of aging and cancer. *Cell Metab.* **35**, 12–35 (2023). 36599298 10.1016/j.cmet.2022.11.001

[43] López-Otín, C., Blasco, M. A., Partridge, L., Serrano, M. & Kroemer, G. Hallmarks of aging: an expanding universe. *Cell* **186**, 243–278 (2023). 36599349 10.1016/j.cell.2022.11.001

[44] Weinert, B. T. & Timiras, P. S. Invited review: theories of aging. *J. Appl. Physiol.* **95**, 1706–1716 (2003). 12970376 10.1152/japplphysiol.00288.2003

[45] Elder Jr, G. H., Johnson, M. K., Crosnoe, R., Mortimer, J. T. & Shanahan, M. J. (eds Mortimer, J. T. & Shanahan, M. J.) Ch. 3–19 (Kluwer Academic/Plenum Publishers, 2003).

[46] Crimmins, E. M. Social hallmarks of aging: suggestions for geroscience research. *Ageing Res. Rev.* **63**, 101136 (2020). 32798771 10.1016/j.arr.2020.101136 PMC7530044

[47] Geronimus, A. T. The weathering hypothesis and the health of African-American women and infants: evidence and speculations. *Ethn. Dis.* **2**, 207–221 (1992). 1467758

[48] Han, X. et al. Cancer history and accelerated aging: findings from a nationally representative sample in the US. *Cancer Causes Control* **36**, 379–388 (2025). 39633205 10.1007/s10552-024-01941-w

[49] Oblak, L., van der Zaag, J., Higgins-Chen, A. T., Levine, M. E. & Boks, M. P. A systematic review of biological, social and environmental factors associated with epigenetic clock acceleration. *Ageing Res. Rev.* **69**, 101348 (2021). 33930583 10.1016/j.arr.2021.101348

[50] Lim, S., Nzegwu, D. & Wright, M. L. The impact of psychosocial stress from life trauma and racial discrimination on epigenetic aging-A systematic review. *Biol. Res. Nurs.* **24**, 202–215 (2022). 35102751 10.1177/10998004211060561 PMC9096197

[51] Carroll, J. E., Bower, J. E. & Ganz, P. A. Cancer-related accelerated ageing and biobehavioural modifiers: a framework for research and clinical care. *Nat. Rev. Clin. Oncol.* **19**, 173–187 (2022). 34873313 10.1038/s41571-021-00580-3 PMC9974153

[52] Petrovic, D. et al. Life-course socioeconomic factors are associated with markers of epigenetic aging in a population-based study. *Psychoneuroendocrinology* **147**, 105976 (2023). 36417838 10.1016/j.psyneuen.2022.105976

[53] Brady, S. S. et al. Applying concepts of life course theory and life course epidemiology to the study of bladder health and lower urinary tract symptoms among girls and women. *Neurourol. Urodyn.* **39**, 1185–1202 (2020). 32119156 10.1002/nau.24325 PMC7659467

[54] Kuh, D., Cooper, R., Hardy, R., Richards, M. & Ben-Shlomo, Y. *A life course approach to healthy ageing*. (OUP Oxford, 2013).

[55] Bengtson, V. L., Elder, G. H. & Putney, N. M. in The *Ca*mbridg*e* Handb*ook of Age and Ageing Cambridge Handbooks in Psychology* (ed. Malcolm, L. J.) 493–501 (Cambridge University Press, 2005).

[56] Mandelblatt, J. S. et al. Applying a life course biological age framework to improving the care of individuals with adult cancers: review and research recommendations. *JAMA Oncol.* **7**, 1692–1699 (2021). 34351358 10.1001/jamaoncol.2021.1160 PMC8602673

[57] Waltz, C. F., Lenz, E. R. & Strickland, O. L. *Measurement in nursing and health research*. 4th edn, (Springer Publishing Company, 2010).

[58] Earp, J. A. & Ennett, S. T. Conceptual models for health education research and practice. *Health Educ. Res.* **6**, 163–171 (1991). 10148689 10.1093/her/6.2.163

[59] Kallen, V. et al. Aging and allostasis: using Bayesian network analytics to explore and evaluate allostatic markers in the context of aging. *Diagnostics* **11**, 157 (2021). 33494482 10.3390/diagnostics11020157 PMC7912325

[60] Horvath, S. DNA methylation age of human tissues and cell types. *Genome Biol.* **14**, R115 (2013). 24138928 10.1186/gb-2013-14-10-r115 PMC4015143

[61] Katz, S., Ford, A. B., Moskowitz, R. W., Jackson, B. A. & Jaffe, M. W. Studies of illness in the aged: the index of ADL: a standardized measure of biological and psychosocial function. *JAMA* **185**, 914–919 (1963). 14044222 10.1001/jama.1963.03060120024016

[62] Karnofsky, D. *Performance scale*. (Plenum Press, New York, 1977).

[63] Hajj, A. et al. Psychometric properties of the 37-item functional assessment of cancer therapy-cognitive function (FACT-Cog) scale. *Future Oncol.* **18**, 3741–3753 (2022). 36345984 10.2217/fon-2022-0438

[64] Williams, A. M. et al. Deficit accumulation index and biological markers of aging in survivors of childhood cancer. *JAMA Netw. Open* **6**, e2344015 (2023). 37983031 10.1001/jamanetworkopen.2023.44015 PMC10660189

[65] Jones, D. M., Song, X. & Rockwood, K. Operationalizing a frailty index from a standardized comprehensive geriatric assessment. *J. Am. Geriatr. Soc.* **52**, 1929–1933 (2004). 15507074 10.1111/j.1532-5415.2004.52521.x

[66] Teschendorff, A. E. & Horvath, S. Epigenetic ageing clocks: statistical methods and emerging computational challenges. *Nat. Rev. Genet.* **26**, 350–368 (2025). 39806006 10.1038/s41576-024-00807-w

[67] Sayed, N. et al. An inflammatory aging clock (iAge) based on deep learning tracks multimorbidity, immunosenescence, frailty and cardiovascular aging. *Nat. Aging* **1**, 598–615 (2021). 34888528 10.1038/s43587-021-00082-y PMC8654267

[68] Argentieri, M. A. et al. Proteomic aging clock predicts mortality and risk of common age-related diseases in diverse populations. *Nat. Med.* **30**, 2450–2460 (2024). 39117878 10.1038/s41591-024-03164-7 PMC11405266

[69] Cole, J. H. & Franke, K. Predicting age using neuroimaging: innovative brain ageing biomarkers. *Trends Neurosci.* **40**, 681–690 (2017). 29074032 10.1016/j.tins.2017.10.001

[70] Marioni, R. E. et al. DNA methylation age of blood predicts all-cause mortality in later life. *Genome Biol.* **16**, 1–12 (2015). 25633388 10.1186/s13059-015-0584-6 PMC4350614

[71] Levine, M. E. et al. An epigenetic biomarker of aging for lifespan and healthspan. *Aging* **10**, 573 (2018). 29676998 10.18632/aging.101414 PMC5940111

[72] Austad, S. N. & Fischer, K. E. Sex differences in lifespan. *Cell Metab.* **23**, 1022–1033 (2016). 27304504 10.1016/j.cmet.2016.05.019 PMC4932837

[73] Hägg, S. & Jylhävä, J. Sex differences in biological aging with a focus on human studies. *Elife* **10**, 10.7554/eLife.63425 (2021). 10.7554/eLife.63425 PMC8118651 33982659

[74] Gordon, E. H. et al. Sex differences in frailty: a systematic review and meta-analysis. *Exp. Gerontol.* **89**, 30–40 (2017). 28043934 10.1016/j.exger.2016.12.021

[75] Unger, J. M. et al. Sex differences in risk of severe adverse events in patients receiving immunotherapy, targeted therapy, or chemotherapy in cancer clinical trials. *J. Clin. Oncol.* **40**, 1474–1486 (2022). 35119908 10.1200/JCO.21.02377 PMC9061143

[76] Koch, M. et al. Gender differences in symptom burden, functional performance and global quality of life of lung cancer patients receiving inpatient versus outpatient treatment. *Cancer Manag. Res.* **15**, 175–183 (2023). 36852345 10.2147/CMAR.S397198 PMC9961146

[77] Miaskowski, C. et al. Latent class analysis reveals distinct subgroups of patients based on symptom occurrence and demographic and clinical characteristics. *J. Pain. Symptom Manag.* **50**, 28–37 (2015). 10.1016/j.jpainsymman.2014.12.011 PMC4492860 25647419

[78] Alberts, S. C. et al. in *Sociality, hierarchy, health: comparative biodemography: a collection of papers* (National Academies Press, 2014). 25254285

[79] Kankaanpää, A. et al. Do epigenetic clocks provide explanations for sex differences in life span? a cross-sectional twin study. *J. Gerontol. A Biol. Sci. Med. Sci.* **77**, 1898–1906 (2022). 34752604 10.1093/gerona/glab337 PMC9434475

[80] Fischer, K. E. & Riddle, N. C. Sex differences in aging: genomic instability. *J. Gerontol. A Biol. Sci. Med. Sci.* **73**, 166–174 (2018). 28575157 10.1093/gerona/glx105 PMC5861920

[81] *Race, class, and gender: Intersections and inequalities*. 11 edn, (Cengage Learning, Inc., 2024).

[82] American Psychological Association. *2015 Stress in America: The Impact of Discrimination*, <https://www.apa.org/news/press/releases/stress/2015/impact> (2015).

[83] Brody, G. H., Miller, G. E., Yu, T., Beach, S. R. & Chen, E. Supportive family environments ameliorate the link between racial discrimination and epigenetic aging: replication across two longitudinal cohorts. *Psychol. Sci.* **27**, 530–541 (2016). 26917213 10.1177/0956797615626703 PMC4833531

[84] Mandelblatt, J. S. et al. Association between major discrimination and deficit accumulation in African American cancer survivors: the Detroit Research on. *Cancer Surviv. Study Cancer* **129**, 1557–1568 (2023). 10.1002/cncr.34673 PMC10568940 36935617

[85] Jain, P. et al. The association of epigenetic age acceleration and multimorbidity at age 90 in the Women’s Health Initiative. *J. Gerontol. A Biol. Sci. Med. Sci.* **78**, 2274–2281 (2023). 36107798 10.1093/gerona/glac190 PMC10692424

[86] Harvanek, Z. M., Fogelman, N., Xu, K. & Sinha, R. Psychological and biological resilience modulates the effects of stress on epigenetic aging. *Transl. Psychiatry* **11**, 601 (2021). 34839356 10.1038/s41398-021-01735-7 PMC8627511

[87] O’Súilleabháin, P. S. et al. Personality pathways to mortality: interleukin-6 links conscientiousness to mortality risk. *Brain Behav. Immun.* **93**, 238–244 (2021). 33571630 10.1016/j.bbi.2021.01.032 PMC7979517

[88] Boyle, C. C., Cole, S. W., Dutcher, J. M., Eisenberger, N. I. & Bower, J. E. Changes in eudaimonic well-being and the conserved transcriptional response to adversity in younger breast cancer survivors. *Psychoneuroendocrinology* **103**, 173–179 (2019). 30703712 10.1016/j.psyneuen.2019.01.024

[89] Turiano, N. A., Chapman, B. P., Gruenewald, T. L. & Mroczek, D. K. Personality and the leading behavioral contributors of mortality. *Health Psychol.* **34**, 51 (2015). 24364374 10.1037/hea0000038 PMC4103968

[90] Bonanno, G. A. Loss, trauma, and human resilience: Have we underestimated the human capacity to thrive after extremelyaversive events? *American Psychologist* **59**, 20–28 (2004). 14736317 10.1037/0003-066X.59.1.20

[91] Zeng, Y. & Shen, K. Resilience significantly contributes to exceptional longevity. *Curr. Gerontol. Geriatr. Res.* **2010**, 525693 (2010). 21197075 10.1155/2010/525693 PMC3004383

[92] Zhang, A. et al. Associations between psychological resilience and epigenetic clocks in the Health and Retirement Study. *Geroscience* **46**, 961–968 (2024). 37707649 10.1007/s11357-023-00940-0 PMC10828333

[93] Lukkahatai, N. et al. Feasibility of DNA methylation age as a biomarker of symptoms and resilience among cancer survivors with multiple chronic conditions. *Biomedicines* **11**, 10.3390/biomedicines11113076 (2023). 10.3390/biomedicines11113076 PMC10669866 38002076

[94] Jakovljevic, K. et al. Higher levels of stress are associated with a significant symptom burden in oncology outpatients receiving chemotherapy. *J. Pain. Symptom Manag.* **61**, 24–31.e24 (2021). 10.1016/j.jpainsymman.2020.07.019 PMC7770050 32721501

[95] Ryff, C. D. Happiness is everything, or is it? Explorations on the meaning of psychological well-being. *J. Personal. Soc. Psychol.* **57**, 1069 (1989).

[96] Kim, E. S., Nakamura, J. S., Strecher, V. J. & Cole, S. W. Reduced epigenetic age in older adults with high sense of purpose in life. *J. Gerontology Ser. A* **78**, 1092–1099 (2023). 10.1093/gerona/glad092 PMC10329221 36966357

[97] Cha, S. E., Song, J., Cole, S. & Ryff, C. D. Cumulative stress and epigenetic aging: examining the role of psychological moderators. *Health Psychol.*10.1037/hea0001524 (2025). 10.1037/hea0001524 PMC12303163 40455542

[98] Lorem, G., Cook, S., Leon, D. A., Emaus, N. & Schirmer, H. Self-reported health as a predictor of mortality: a cohort study of its relation to other health measurements and observation time. *Sci. Rep.* **10**, 4886 (2020). 32184429 10.1038/s41598-020-61603-0 PMC7078209

[99] Williams, K., Jackson, S. E., Beeken, R. J., Steptoe, A. & Wardle, J. The impact of a cancer diagnosis on health and well-being: a prospective, population-based study. *Psycho-Oncology* **25**, 626–632 (2016). 26425927 10.1002/pon.3998 PMC4915489

[100] Moser, N. et al. Association between self-reported and objectively assessed physical functioning in the general population. *Sci. Rep.* **14**, 16236 (2024). 39004682 10.1038/s41598-024-64939-z PMC11247090

[101] Christian, L. M. et al. Poorer self-rated health is associated with elevated inflammatory markers among older adults. *Psychoneuroendocrinology* **36**, 1495–1504 (2011). 21601365 10.1016/j.psyneuen.2011.04.003 PMC3161147

[102] Jylhä, M. What is self-rated health and why does it predict mortality? Towards a unified conceptual model. *Soc. Sci. Med.* **69**, 307–316 (2009). 19520474 10.1016/j.socscimed.2009.05.013

[103] Li, D. L. et al. Self-rated health, epigenetic ageing, and long-term mortality in older Australians. *GeroScience* **46**, 5505–5515 (2024). 38795183 10.1007/s11357-024-01211-2 PMC11493901

[104] Rouland, A. et al. Personality types in individuals with type 1 and type 2 diabetes. *Endocr. Connect* **9**, 254–260 (2020). 32101526 10.1530/EC-19-0499 PMC7077523

[105] Jokela, M. et al. Personality and all-cause mortality: individual-participant meta-analysis of 3,947 deaths in 76,150 adults. *Am. J. Epidemiol.* **178**, 667–675 (2013). 23911610 10.1093/aje/kwt170 PMC3755650

[106] Graham, E. K. et al. Personality predicts mortality risk: an integrative data analysis of 15 international longitudinal studies. *J. Res. Personal.* **70**, 174–186 (2017). 10.1016/j.jrp.2017.07.005 PMC5722274 29230075

[107] Lyons, C. E., Razzoli, M. & Bartolomucci, A. The impact of life stress on hallmarks of aging and accelerated senescence: connections in sickness and in health. *Neurosci. Biobehav. Rev.* **153**, 105359 (2023). 37586578 10.1016/j.neubiorev.2023.105359 PMC10592082

[108] Kruk, J., Aboul-Enein, B. H., Bernstein, J. & Gronostaj, M. Psychological stress and cellular aging in cancer: a meta-analysis. *Oxid. Med. Cell Longev.* **2019**, 1270397 (2019). 31814865 10.1155/2019/1270397 PMC6877941

[109] Wu, Z., Qu, J., Zhang, W. & Liu, G. H. Stress, epigenetics, and aging: unraveling the intricate crosstalk. *Mol. Cell* **84**, 34–54 (2023). 37963471 10.1016/j.molcel.2023.10.006

[110] Zannas, A. S. Epigenetics as a key link between psychosocial stress and aging: concepts, evidence, mechanisms. *Dialogues Clin. Neurosci.* **21**, 389–396 (2019). 31949406 10.31887/DCNS.2019.21.4/azannas PMC6952744

[111] Wolf, E. J. & Morrison, F. G. Traumatic stress and accelerated cellular aging: from epigenetics to cardiometabolic disease. *Curr. Psychiatry Rep.* **19**, 75 (2017). 28852965 10.1007/s11920-017-0823-5 PMC5588711

[112] Qin, T. et al. Stress hormones: unveiling the role in accelerated cellular senescence. *Aging Dis.* **16**, 1946–1970 (2024). 39226159 10.14336/AD.2024.0262 PMC12221407

[113] Wolf, E. J. et al. Traumatic stress and accelerated DNA methylation age: a meta-analysis. *Psychoneuroendocrinology* **92**, 123–134 (2018). 29452766 10.1016/j.psyneuen.2017.12.007 PMC5924645

[114] Zannas, A. S. et al. Lifetime stress accelerates epigenetic aging in an urban, African American cohort: relevance of glucocorticoid signaling. *Genome Biol.* **16**, 266 (2015). 26673150 10.1186/s13059-015-0828-5 PMC4699359

[115] Jovanovic, T. et al. Exposure to violence accelerates epigenetic aging in children. *Sci. Rep.* **7**, 8962 (2017). 28827677 10.1038/s41598-017-09235-9 PMC5566406

[116] Sumner, J. A., Colich, N. L., Uddin, M., Armstrong, D. & McLaughlin, K. A. Early experiences of threat, but not deprivation, are associated with accelerated biological aging in children and adolescents. *Biol. Psychiatry* **85**, 268–278 (2019). 30391001 10.1016/j.biopsych.2018.09.008 PMC6326868

[117] Zannas, A. S. et al. Epigenetic aging and PTSD outcomes in the immediate aftermath of trauma. *Psychol. Med.* **53**, 7170–7179 (2023). 36951141 10.1017/S0033291723000636

[118] Langford, D. J. et al. Distinct stress profiles among oncology patients undergoing chemotherapy. *J. Pain. Symptom Manag.* **59**, 646–657 (2020). 10.1016/j.jpainsymman.2019.10.025 31711968

[119] Ma, J. et al. The association between dietary nutrient intake and acceleration of aging: evidence from NHANES. *Nutrients* **16**, 1635 (2024). 10.3390/nu16111635 PMC11174358 38892569

[120] Leitão, C. et al. The effect of nutrition on aging-a systematic review focusing on aging-related biomarkers. *Nutrients* **14**, 10.3390/nu14030554 (2022). 10.3390/nu14030554 PMC8838212 35276919

[121] Yang, Y. et al. Smoking-related DNA methylation is associated with DNA methylation phenotypic age acceleration: the Veterans Affairs Normative Aging Study. *Int. J. Environ. Res. Public Health* **16**, 10.3390/ijerph16132356 (2019). 10.3390/ijerph16132356 PMC6651499 31277270

[122] Xiao, C. et al. Association of epigenetic age acceleration with risk factors, survival, and quality of life in patients with head and neck cancer. *Int. J. Radiat. Oncol. Biol. Phys.* **111**, 157–167 (2021). 33882281 10.1016/j.ijrobp.2021.04.002 PMC8802868

[123] Lu, A. T. et al. DNA methylation GrimAge strongly predicts lifespan and healthspan. *Aging* **11**, 303 (2019). 30669119 10.18632/aging.101684 PMC6366976

[124] Dev, R. et al. Coping strategies and associated symptom burden among patients with advanced cancer. *Oncologist* **29**, 166–175 (2023). 10.1093/oncolo/oyad253 PMC10836315 37669020

[125] Calvo-Schimmel, A. et al. Stress and coping in patients with cancer with depression and sleep disturbance. *Oncol. Nurs. Forum* **51**, 243–262 (2024). 38668910 10.1188/24.ONF.243-262

[126] Nyembwe, A. et al. Discrimination, coping, and DNA accelerated aging among African American mothers of the InterGEN study. *Epigenomes* **9**, 14 (2025). 40407423 10.3390/epigenomes9020014 PMC12101303

[127] Xu, M. et al. Senolytics improve physical function and increase lifespan in old age. *Nat. Med.* **24**, 1246–1256 (2018). 29988130 10.1038/s41591-018-0092-9 PMC6082705

[128] Jaskelioff, M. et al. Telomerase reactivation reverses tissue degeneration in aged telomerase-deficient mice. *Nature* **469**, 102–106 (2011). 21113150 10.1038/nature09603 PMC3057569

[129] Cassidy, L. D. et al. Temporal inhibition of autophagy reveals segmental reversal of ageing with increased cancer risk. *Nat. Commun.* **11**, 307 (2020). 31949142 10.1038/s41467-019-14187-x PMC6965206

[130] Hanahan, D. & Weinberg, R. A. The hallmarks of cancer. *cell* **100**, 57–70 (2000). 10647931 10.1016/s0092-8674(00)81683-9

[131] Wang, S., Prizment, A., Thyagarajan, B. & Blaes, A. Cancer treatment-induced accelerated aging in cancer survivors: biology and assessment. *Cancers* **13**10.3390/cancers13030427 (2021). 10.3390/cancers13030427 PMC7865902 33498754

[132] Abraham, S. et al. Accelerated aging in cancer and cancer treatment: current status of biomarkers. *Cancer Med.* **14**, e70929 (2025). 40322791 10.1002/cam4.70929 PMC12051034

[133] Tenchov, R., Sasso, J. M., Wang, X. & Zhou, Q. A. Aging hallmarks and progression and age-related diseases: a landscape view of research advancement. *ACS Chem. Neurosci.* **15**, 1–30 (2024). 38095562 10.1021/acschemneuro.3c00531 PMC10767750

[134] Montégut, L., López-Otín, C. & Kroemer, G. Aging and cancer. *Mol. Cancer* **23**, 106 (2024). 38760832 10.1186/s12943-024-02020-z PMC11102267

[135] Vijg, J. & Suh, Y. Genome instability and aging. *Annu Rev. Physiol.* **75**, 645–668 (2013). 23398157 10.1146/annurev-physiol-030212-183715

[136] Clarke, T. L. & Mostoslavsky, R. DNA repair as a shared hallmark in cancer and ageing. *Mol. Oncol.* **16**, 3352–3379 (2022). 35834102 10.1002/1878-0261.13285 PMC9490147

[137] Paniagua, I. & Jacobs, J. J. L. Freedom to err: the expanding cellular functions of translesion DNA polymerases. *Mol. Cell* **83**, 3608–3621 (2023). 37625405 10.1016/j.molcel.2023.07.008

[138] Gyenis, A. et al. Genome-wide RNA polymerase stalling shapes the transcriptome during aging. *Nat. Genet.* **55**, 268–279 (2023). 36658433 10.1038/s41588-022-01279-6 PMC9925383

[139] Soheili-Nezhad, S., Ibáñez-Solé, O., Izeta, A., Hoeijmakers, J. H. J. & Stoeger, T. Time is ticking faster for long genes in aging. *Trends Genet.* **40**, 299–312 (2024). 38519330 10.1016/j.tig.2024.01.009 PMC11003850

[140] Schumacher, B., Pothof, J., Vijg, J. & Hoeijmakers, J. H. J. The central role of DNA damage in the ageing process. *Nature* **592**, 695–703 (2021). 33911272 10.1038/s41586-021-03307-7 PMC9844150

[141] Karikkineth, A. C., Scheibye-Knudsen, M., Fivenson, E., Croteau, D. L. & Bohr, V. A. Cockayne syndrome: clinical features, model systems and pathways. *Ageing Res. Rev.* **33**, 3–17 (2017). 27507608 10.1016/j.arr.2016.08.002 PMC5195851

[142] Tilsed, C. M., Fisher, S. A., Nowak, A. K., Lake, R. A. & Lesterhuis, W. J. Cancer chemotherapy: insights into cellular and tumor microenvironmental mechanisms of action. *Front. Oncol.* **12**, 960317 (2022). 35965519 10.3389/fonc.2022.960317 PMC9372369

[143] Taylor, A. M. et al. PARP (Poly ADP-Ribose Polymerase) inhibitors for locally advanced or metastatic breast cancer. *Cochrane Database Syst. Rev.* **4**, Cd011395 (2021). 33886122 10.1002/14651858.CD011395.pub2 PMC8092476

[144] Turner, K. J., Vasu, V. & Griffin, D. K. Telomere biology and human phenotype. *Cells* **8**10.3390/cells8010073 (2019). 10.3390/cells8010073 PMC6356320 30669451

[145] Martínez, P. & Blasco, M. A. Telomere-driven diseases and telomere-targeting therapies. *J. Cell Biol.* **216**, 875 (2017). 28254828 10.1083/jcb.201610111 PMC5379954

[146] Gallicchio, L., Gadalla, S. M., Murphy, J. D. & Simonds, N. I. The effect of cancer treatments on telomere length: a systematic review of the literature. *J. Natl. Cancer Inst.* **110**, 1048–1058 (2018). 30272225 10.1093/jnci/djy189 PMC6186521

[147] Hayashi, M. T., Cesare, A. J., Fitzpatrick, J. A. J., Lazzerini-Denchi, E. & Karlseder, J. A telomere-dependent DNA damage checkpoint induced by prolonged mitotic arrest. *Nat. Struct. Mol. Biol.* **19**, 387–394 (2012). 22407014 10.1038/nsmb.2245 PMC3319806

[148] Szyper-Kravitz, M. et al. Granulocyte colony-stimulating factor administration upregulates telomerase activity in CD34+ haematopoietic cells and may prevent telomere attrition after chemotherapy. *Br. J. Haematol.* **120**, 329–336 (2003). 12542495 10.1046/j.1365-2141.2003.04043.x

[149] El Zarif, T. et al. TERT promoter mutations frequency across race, sex, and cancer type. *Oncologist* **29**, 8–14 (2024). 37462445 10.1093/oncolo/oyad208 PMC10769781

[150] Shammas, M. A. Telomeres, lifestyle, cancer, and aging. *Curr. Opin. Clin. Nutr. Metab. Care* **14**, 28–34 (2011). 21102320 10.1097/MCO.0b013e32834121b1 PMC3370421

[151] Vaiserman, A. & Krasnienkov, D. Telomere length as a marker of biological age: state-of-the-art, open issues, and future perspectives. *Front. Genet.* **11**, 630186 (2021). 33552142 10.3389/fgene.2020.630186 PMC7859450

[152] Toraño, E. G., García, M. G., Fernández-Morera, J. L., Niño-García, P. & Fernández, A. F. The impact of external factors on the epigenome: in utero and over lifetime. *Biomed. Res. Int.* **2016**, 2568635 (2016). 27294112 10.1155/2016/2568635 PMC4887632

[153] Hannum, G. et al. Genome-wide methylation profiles reveal quantitative views of human aging rates. *Mol. cell* **49**, 359–367 (2013). 23177740 10.1016/j.molcel.2012.10.016 PMC3780611

[154] Lu, A. T. et al. DNA methylation GrimAge version 2. *Aging* **14**, 9484–9549 (2022). 36516495 10.18632/aging.204434 PMC9792204

[155] Dugué, P. A. et al. DNA methylation-based biological aging and cancer risk and survival: pooled analysis of seven prospective studies. *Int. J. Cancer* **142**, 1611–1619 (2018). 29197076 10.1002/ijc.31189

[156] Fransquet, P. D., Wrigglesworth, J., Woods, R. L., Ernst, M. E. & Ryan, J. The epigenetic clock as a predictor of disease and mortality risk: a systematic review and meta-analysis. *Clin. Epigenetics* **11**, 62 (2019). 30975202 10.1186/s13148-019-0656-7 PMC6458841

[157] Horvath, S. & Ritz, B. R. Increased epigenetic age and granulocyte counts in the blood of Parkinson’s disease patients. *Aging* **7**, 1130–1142 (2015). 26655927 10.18632/aging.100859 PMC4712337

[158] Roetker, N. S., Pankow, J. S., Bressler, J., Morrison, A. C. & Boerwinkle, E. Prospective study of epigenetic age acceleration and incidence of cardiovascular disease outcomes in the aric study (Atherosclerosis Risk in Communities. *Circ. Genom. Precis.Med* **11**, e001937 (2018). 29555670 10.1161/CIRCGEN.117.001937 PMC5863591

[159] Sehl, M. E., Carroll, J. E., Horvath, S. & Bower, J. E. The acute effects of adjuvant radiation and chemotherapy on peripheral blood epigenetic age in early stage breast cancer patients. *NPJ Breast Cancer* **6**, 23 (2020). 32566744 10.1038/s41523-020-0161-3 PMC7293278

[160] Yang, G. S. et al. Exploring the relationship between DNA methylation age measures and psychoneurological symptoms in women with early-stage breast cancer. *Support Care Cancer* **31**, 65 (2022). 36538110 10.1007/s00520-022-07519-z

[161] Labbadia, J. & Morimoto, R. I. The biology of proteostasis in aging and disease. *Annu. Rev. Biochem.* **84**, 435–464 (2015). 25784053 10.1146/annurev-biochem-060614-033955 PMC4539002

[162] Lipinski, M. M. et al. Genome-wide analysis reveals mechanisms modulating autophagy in normal brain aging and in Alzheimer’s disease. *Proc. Natl. Acad. Sci. USA* **107**, 14164–14169 (2010). 20660724 10.1073/pnas.1009485107 PMC2922576

[163] Chen, L. et al. Enhanced degradation of misfolded proteins promotes tumorigenesis. *Cell Rep.* **18**, 3143–3154 (2017). 28355566 10.1016/j.celrep.2017.03.010 PMC5603913

[164] Nagaraj, N. S., Singh, O. V. & Merchant, N. B. Proteomics: a strategy to understand the novel targets in protein misfolding and cancer therapy. *Expert Rev. Proteom.* **7**, 613–623 (2010). 10.1586/epr.10.70 PMC4339030 20653514

[165] Ho Zhi Guang, M. et al. Targeting proteotoxic stress in cancer: a review of the role that protein quality control pathways play in oncogenesis. *Cancers* **11**, 66 (2019). 30634515 10.3390/cancers11010066 PMC6356294

[166] Wang, S. et al. Proteomic aging clocks and the risk of mortality among longer-term cancer survivors in the Atherosclerosis Risk in Communities (ARIC) Study. *medRxiv*10.1101/2024.07.09.24309726 (2024).

[167] Morales-Valencia, J. & David, G. The contribution of physiological and accelerated aging to cancer progression through senescence-induced inflammation. *Front. Oncol.* **11**, 747822 (2021). 34621683 10.3389/fonc.2021.747822 PMC8490756

[168] Hoare, M. & Narita, M. Transmitting senescence to the cell neighbourhood. *Nat. cell Biol.* **15**, 887–889 (2013). 23907191 10.1038/ncb2811

[169] Liu, Y. et al. Expression of p16(INK4a) in peripheral blood T-cells is a biomarker of human aging. *Aging Cell* **8**, 439–448 (2009). 19485966 10.1111/j.1474-9726.2009.00489.x PMC2752333

[170] Wood, W. A. et al. Chemotherapy and stem cell transplantation increase p16(INK4a) expression, a biomarker of T-cell aging. *EBioMedicine* **11**, 227–238 (2016). 27591832 10.1016/j.ebiom.2016.08.029 PMC5049997

[171] Rosko, A. et al. Autologous hematopoietic stem cell transplant induces the molecular aging of T-cells in multiple myeloma. *Bone Marrow Transpl.* **50**, 1379–1381 (2015). 10.1038/bmt.2015.143 PMC4821192 26121107

[172] Smitherman, A. B. et al. Accelerated aging among childhood, adolescent, and young adult. *Cancer* **126**, 4975–4983 (2020). 32830315 10.1002/cncr.33112 PMC7607511

[173] Sanoff, H. K. et al. Effect of cytotoxic chemotherapy on markers of molecular age in patients with breast cancer. *J. Natl. Cancer Inst.* **106**, dju057 (2014). 24681605 10.1093/jnci/dju057 PMC3982894

[174] Conover, C. A. & Oxvig, C. The IGF system and aging. *Endocr. Rev.* **46**, 214–223 (2024). 10.1210/endrev/bnae029 PMC11894535 39418083

[175] Mao, Y., Xia, Z., Xia, W. & Jiang, P. Metabolic reprogramming, sensing, and cancer therapy. *Cell Rep.* **43**, 115064 (2024). 39671294 10.1016/j.celrep.2024.115064

[176] Lee, J. S., Tocheny, C. E. & Shaw, L. M. The insulin-like growth factor signaling pathway in breast cancer: an elusive therapeutic target. *Life* **12**, 10.3390/life12121992 (2022). 10.3390/life12121992 PMC9782138 36556357

[177] Kabacik, S. et al. The relationship between epigenetic age and the hallmarks of aging in human cells. *Nat. Aging* **2**, 484–493 (2022). 37034474 10.1038/s43587-022-00220-0 PMC10077971

[178] Kudryavtseva, A. V. et al. Mitochondrial dysfunction and oxidative stress in aging and cancer. *Oncotarget* **7**, 44879–44905 (2016). 27270647 10.18632/oncotarget.9821 PMC5216692

[179] Sanchez-Contreras, M. & Kennedy, S. R. The complicated nature of somatic mtDNA mutations in aging. *Front. Aging***ume 2**, 2021 (2022). 10.3389/fragi.2021.805126 PMC8896747 35252966

[180] Bartman, S., Coppotelli, G. & Ross, J. M. Mitochondrial dysfunction: a key player in brain aging and diseases. *Curr. Issues Mol. Biol.* **46**, 1987–2026 (2024). 38534746 10.3390/cimb46030130 PMC10969191

[181] Jiang, H. et al. Drug-induced oxidative stress in cancer treatments: angel or devil. *Redox Biol.* **63**, 102754 (2023). 37224697 10.1016/j.redox.2023.102754 PMC10220276

[182] Songbo, M. et al. Oxidative stress injury in doxorubicin-induced cardiotoxicity. *Toxicol. Lett.* **307**, 41–48 (2019). 30817977 10.1016/j.toxlet.2019.02.013

[183] Zhang, C., Xu, C., Gao, X. & Yao, Q. Platinum-based drugs for cancer therapy and anti-tumor strategies. *Theranostics* **12**, 2115–2132 (2022). 35265202 10.7150/thno.69424 PMC8899578

[184] Mitry, M. A. et al. Accelerated cardiomyocyte senescence contributes to late-onset doxorubicin-induced cardiotoxicity. *Am. J. Physiol. Cell Physiol.* **318**, C380–c391 (2020). 31913702 10.1152/ajpcell.00073.2019 PMC7052608

[185] Linders, A. N. et al. A review of the pathophysiological mechanisms of doxorubicin-induced cardiotoxicity and aging. *npj Aging* **10**, 9 (2024). 38263284 10.1038/s41514-024-00135-7 PMC10806194

[186] Zhu, X. et al. Inflammation, epigenetics, and metabolism converge to cell senescence and ageing: the regulation and intervention. *Signal Transduct. Target Ther.* **6**, 245 (2021). 34176928 10.1038/s41392-021-00646-9 PMC8236488

[187] Furman, D. et al. Chronic inflammation in the etiology of disease across the life span. *Nat. Med.* **25**, 1822–1832 (2019). 31806905 10.1038/s41591-019-0675-0 PMC7147972

[188] Alfano, C. M. et al. Inflammatory cytokines and comorbidity development in breast cancer survivors versus noncancer controls: evidence for accelerated aging. *J. Clin. Oncol.* **35**, 149–156 (2017). 27893337 10.1200/JCO.2016.67.1883 PMC5455675

[189] Biragyn, A. & Ferrucci, L. Gut dysbiosis: a potential link between increased cancer risk in ageing and inflammaging. *Lancet Oncol.* **19**, e295–e304 (2018). 29893261 10.1016/S1470-2045(18)30095-0 PMC6047065

[190] Zou, S., Fang, L. & Lee, M.-H. Dysbiosis of gut microbiota in promoting the development of colorectal cancer. *Gastroenterol. Rep.* **6**, 1–12 (2017). 10.1093/gastro/gox031 PMC5806407 29479437

[191] Biagi, E. et al. Through ageing, and beyond: gut microbiota and inflammatory status in seniors and centenarians. *PloS one* **5**, e10667 (2010). 20498852 10.1371/journal.pone.0010667 PMC2871786

[192] Guevara-Ramírez, P. et al. Gut microbiota disruption in hematologic cancer therapy: molecular insights and implications for treatment efficacy. *Int. J. Mol. Sci.* **25**10.3390/ijms251910255 (2024). 10.3390/ijms251910255 PMC11476909 39408584

[193] Wen, N. N., Sun, L. W., Geng, Q. & Zheng, G. H. Gut microbiota changes associated with frailty in older adults: a systematic review of observational studies. *World J. Clin. Cases* **12**, 6815–6825 (2024). 39687638 10.12998/wjcc.v12.i35.6815 PMC11525918

[194] Jemimah, S., Chabib, C. M. M., Hadjileontiadis, L. & AlShehhi, A. Gut microbiome dysbiosis in Alzheimer’s disease and mild cognitive impairment: a systematic review and meta-analysis. *PLoS ONE* **18**, e0285346 (2023). 37224131 10.1371/journal.pone.0285346 PMC10208513

[195] Coradduzza, D. et al. Age-related cognitive decline, focus on microbiome: a systematic review and meta-analysis. *Int. J. Mol. Sci.* **24**, 13680 (2023). 37761988 10.3390/ijms241813680 PMC10531012

[196] Sharma, S. et al. Association between accelerated biological aging, diet, and gut microbiome. *Microorganisms* **12**, 1719 (2024). 39203561 10.3390/microorganisms12081719 PMC11357197

[197] Torma, F. et al. Alterations of the gut microbiome are associated with epigenetic age acceleration and physical fitness. *Aging Cell* **23**10.1111/acel.14101 (2024). 10.1111/acel.14101 PMC11019127 38414315

[198] Singh, S. et al. Distinct intestinal microbial signatures linked to accelerated systemic and intestinal biological aging. *Microbiome* **12**, 31 (2024). 38383483 10.1186/s40168-024-01758-4 PMC10882811

[199] Cordeiro, A. et al. Late events after treatment with CD19-targeted chimeric antigen receptor modified T cells. *Biol. Blood Marrow Transplant.* **26**, 26–33 (2020). 31419568 10.1016/j.bbmt.2019.08.003 PMC6953906

[200] Juluri, K. R. et al. Severe cytokine release syndrome is associated with hematologic toxicity following CD19 CAR T-cell therapy. *Blood Adv.* **6**, 2055–2068 (2022). 34666344 10.1182/bloodadvances.2020004142 PMC9006285

[201] Demaria, M. et al. An essential role for senescent cells in optimal wound healing through secretion of PDGF-AA. *Dev. Cell* **31**, 722–733 (2014). 25499914 10.1016/j.devcel.2014.11.012 PMC4349629

[202] Cheung, Y.-M., Ramchand, S. K., Yeo, B. & Grossmann, M. Cardiometabolic effects of endocrine treatment of estrogen receptor–positive early breast cancer. *J. Endocr. Soc.* **3**, 1283–1301 (2019). 31259291 10.1210/js.2019-00096 PMC6595530

[203] min, H.-Y. & Lee, H.-Y. Molecular targeted therapy for anticancer treatment. *Exp. Mol. Med.* **54**, 1670–1694 (2022). 36224343 10.1038/s12276-022-00864-3 PMC9636149

[204] Nguyen, S. M. et al. Chemotherapy-induced toxicities and their associations with clinical and non-clinical factors among breast cancer patients in Vietnam. *Curr. Oncol.* **29**, 8269–8284 (2022). 36354713 10.3390/curroncol29110653 PMC9689154

[205] Chang, W. H., Neal, R. D., Forster, M. D. & Lai, A. G. Cumulative burden of 144 conditions, critical care hospitalisation and premature mortality across 26 adult cancers. *Nat. Commun.* **14**, 1484 (2023). 36932095 10.1038/s41467-023-37231-3 PMC10023774

[206] Wang, C. et al. Accelerated aging associated with cancer characteristics and treatments among breast cancer survivors. *Aging* **17**, 643–656 (2025). 40163417 10.18632/aging.206218 PMC11984420

[207] McGowan, J. V. et al. Anthracycline chemotherapy and cardiotoxicity. *Cardiovasc. Drugs Ther.* **31**, 63–75 (2017). 28185035 10.1007/s10557-016-6711-0 PMC5346598

[208] Bhatia, R., Holtan, S., Jurdi, N. E., Prizment, A. & Blaes, A. Do cancer and cancer treatments accelerate aging. *Curr. Oncol. Rep.* **24**, 1401–1412 (2022). 35796942 10.1007/s11912-022-01311-2 PMC9606015

[209] Kim, J.-E. E., Dodd, M. J., Aouizerat, B. E., Jahan, T. & Miaskowski, C. A review of the prevalence and impact of multiple symptoms in oncology patients. *J. Pain. Symptom Manag.* **37**, 715–736 (2009). 10.1016/j.jpainsymman.2008.04.018 PMC2688644 19019626

[210] Morse, L. et al. Stability and consistency of symptom clusters in younger versus older patients receiving chemotherapy. *BMC Geriatr.* **24**, 164 (2024). 38365584 10.1186/s12877-024-04755-2 PMC10870638

[211] Harris, C. et al. Impact of multimorbidity on symptom burden and symptom clusters in patients receiving chemotherapy. *Cancer Med.* **14**, e70418 (2025). 39910913 10.1002/cam4.70418 PMC11799588

[212] Pandya, C. et al. Association between symptom burden and physical function in older patients with cancer. *J. Am. Geriatr. Soc.* **67**, 998–1004 (2019). 30848838 10.1111/jgs.15864 PMC7851835

[213] Miaskowski, C. et al. Determination of cutpoints for symptom burden in oncology patients receiving chemotherapy. *J. Pain. Symptom Manag.* **63**, 42–51 (2022). 10.1016/j.jpainsymman.2021.07.018 PMC10791137 34333099

[214] Portenoy, R. K. et al. The memorial symptom assessment scale: an instrument for the evaluation of symptom prevalence, characteristics and distress. *Eur. J. Cancer* **30**, 1326–1336 (1994). 10.1016/0959-8049(94)90182-1 7999421

[215] Duan, L., Cui, H., Zhang, W. & Wu, S. Symptoms and experiences of frailty in lung cancer patients with chemotherapy: a mixed-method approach. *Front. Oncol.* **12**, 1019006 (2022). 36276107 10.3389/fonc.2022.1019006 PMC9582838

[216] Hajjar, J. et al. Associations between the gut microbiome and fatigue in cancer patients. *Sci. Rep.* **11**, 5847 (2021). 33712647 10.1038/s41598-021-84783-9 PMC7954807

[217] Harris, C. S. et al. Symptom clusters in patients receiving chemotherapy: a systematic review. *BMJ Support Palliat. Care* **12**, 10–21 (2022). 34921000 10.1136/bmjspcare-2021-003325 PMC8857036

[218] Aktas, A., Walsh, D. & Rybicki, L. Symptom clusters and prognosis in advanced cancer. *Support Care Cancer* **20**, 2837–2843 (2012). 22361827 10.1007/s00520-012-1408-9

[219] Bourassa, K. J. et al. Posttraumatic stress disorder, trauma, and accelerated biological aging among post-9/11 veterans. *Transl. Psychiatry* **14**, 4 (2024). 38184702 10.1038/s41398-023-02704-y PMC10771513

[220] Ryff, C. D. Psychological well-being revisited: advances in the science and practice of eudaimonia. *Psychother. Psychosom.* **83**, 10–28 (2013). 24281296 10.1159/000353263 PMC4241300

[221] Jackson, P., Kempf, M. C., Goodin, B. R., B, A. H. & Aroke, E. N. Neighborhood environment and epigenetic age: a scoping review. *West J. Nurs. Res.* **45**, 1139–1149 (2023). 37902222 10.1177/01939459231208304 PMC10748459

[222] Wood, N. M., Trebilco, T. & Cohen-Woods, S. Scars of childhood socioeconomic stress: a systematic review. *Neurosci. Biobehav. Rev.* **118**, 397–410 (2020). 32795493 10.1016/j.neubiorev.2020.08.001

[223] Sun, F.-K., Lu, C.-Y., Yao, Y. & Chiang, C.-Y. Social functioning, depression, and quality of life among breast cancer patients: a path analysis. *Eur. J. Oncol. Nurs.* **62**, 102237 (2023). 36455513 10.1016/j.ejon.2022.102237

[224] Meier, A. et al. Role functioning is associated with survival in patients with hepatocellular carcinoma. *Qual. Life Res.* **24**, 1669–1675 (2015). 25502092 10.1007/s11136-014-0895-1 PMC4466090

[225] Cabling, M. L., Drago, F., Turner, J., Hurtado-de-Mendoza, A. & Sheppard, V. B. Revisiting agency and medical health technology: actor network theory and breast cancer survivors’ perspectives on an adherence tool. *Health Technol.* **12**, 1071–1084 (2022). 10.1007/s12553-022-00707-1 PMC9660207 36406185

[226] Gregory, K. et al. Prevalence of health behaviors among cancer survivors in the United States. *J. Cancer Surviv* **18**, 1042–1050 (2024). 36933085 10.1007/s11764-023-01347-8 PMC10024006

[227] Wadsworth, M. E. Development of maladaptive coping: a functional adaptation to chronic, uncontrollable stress. *Child Dev. Perspect.* **9**, 96–100 (2015). 26019717 10.1111/cdep.12112 PMC4442090

[228] Gonzales, N. A., Tein, J.-Y., Sandler, I. N. & Friedman, R. J. On the limits of coping:interaction between stress and coping for inner-city adolescents. *J. Adolesc. Res.* **16**, 372–395 (2001).

[229] Solberg, M. A., Peters, R. M., Resko, S. M. & Templin, T. N. Does coping mediate the relationship between adverse childhood experiences and health outcomes in young adults. *J. Child Adolesc. Trauma* **16**, 615–627 (2023). 10.1007/s40653-023-00527-z PMC9944421 36844997

[230] Kornblith, A. B. et al. Long-term psychosocial adjustment of older vs younger survivors of breast and endometrial cancer. *Psycho-Oncology: Journal of the Psychological*. *Soc. Behav. Dimens. Cancer* **16**, 895–903 (2007). 10.1002/pon.1146 17245695

[231] Martins-Klein, B., Bamonti, P. M., Owsiany, M., Naik, A. & Moye, J. Age differences in cancer-related stress, spontaneous emotion regulation, and emotional distress. *Aging Ment. Health* **25**, 250–259 (2021). 31851838 10.1080/13607863.2019.1693972 PMC7299731

[232] Hernández, R. et al. Differences in coping strategies among young adults and the elderly with cancer. *Psychogeriatrics* **19**, 426–434 (2019). 30723983 10.1111/psyg.12420

[233] Husson, O., Zebrack, B. J., Aguilar, C., Hayes-Lattin, B. & Cole, S. Cancer in adolescents and young adults: who remains at risk of poor social functioning over time? *Cancer* **123**, 2743–2751 (2017). 28319256 10.1002/cncr.30656

[234] Cohen, M., Baziliansky, S. & Beny, A. The association of resilience and age in individuals with colorectal cancer: an exploratory cross-sectional study. *J. Geriatr. Oncol.* **5**, 33–39 (2014). 24484716 10.1016/j.jgo.2013.07.009

[235] Matzka, M. et al. Relationship between resilience, psychological distress and physical activity in cancer patients: a cross-sectional observation study. *PLoS ONE* **11**, e0154496 (2016). 27124466 10.1371/journal.pone.0154496 PMC4849643

[236] Meng, X. & D’Arcy, C. Determinants of self-rated health among Canadian seniors over time: a longitudinal population-based study. *Soc. Indic. Res.* **126**, 1343–1353 (2016).

[237] Spuling, S. M., Wolff, J. K. & Wurm, S. Response shift in self-rated health after serious health events in old age. *Soc. Sci. Med.* **192**, 85–93 (2017). 28963988 10.1016/j.socscimed.2017.09.026

[238] Choi, J. H. & Miyamoto, Y. Cultural differences in self-rated health: the role of influence and adjustment. *Jpn. Psychol. Res.* **64**, 156–169 (2022).

[239] Lazarus, R. S. & Folkman, S. *Stress, Appraisal, and Coping*. (Springer Publishing Company, 1984).

[240] Kuh, D., Ben-Shlomo, Y., Lynch, J., Hallqvist, J. & Power, C. Life course epidemiology. *J. Epidemiol. Community Health* **57**, 778 (2003). 14573579 10.1136/jech.57.10.778 PMC1732305

[241] Guida, J. L. et al. Associations of seven measures of biological age acceleration with frailty and all-cause mortality among adult survivors of childhood cancer in the St. Jude Lifetime Cohort. *Nat. Cancer* **5**, 731–741 (2024). 38553617 10.1038/s43018-024-00745-w PMC11139608

[242] Rentscher, K. E. et al. Epigenetic aging in older breast cancer survivors and noncancer controls: preliminary findings from the thinking and living with cancer study. *Cancer* **129**, 2741–2753 (2023). 37259669 10.1002/cncr.34818 PMC10659047

[243] Subica, A. M. & Link, B. G. Cultural trauma as a fundamental cause of health disparities. *Soc. Sci. Med.* **292**, 114574 (2022). 34808396 10.1016/j.socscimed.2021.114574 PMC9006767

[244] Fox, M., Thayer, Z. & Wadhwa, P. D. Acculturation and health: the moderating role of socio-cultural context. *Am. Anthropol.* **119**, 405–421 (2017). 28966344 10.1111/aman.12867 PMC5617140

[245] Lawrence, K. G. et al. Association of neighborhood deprivation with epigenetic aging using 4 clock metrics. *JAMA Netw. Open* **3**, e2024329–e2024329 (2020). 33146735 10.1001/jamanetworkopen.2020.24329 PMC7643028

[246] Martin, C. L. et al. Neighborhood environment, social cohesion, and epigenetic aging. *Aging* **13**, 7883–7899 (2021). 33714950 10.18632/aging.202814 PMC8034890

[247] Roth, D. L., Fredman, L. & Haley, W. E. Informal caregiving and its impact on health: a reappraisal from population-based studies. *Gerontologist* **55**, 309–319 (2015). 26035608 10.1093/geront/gnu177 PMC6584119

[248] Christian, L. M. et al. Understanding the health effects of caregiving stress: new directions in molecular aging. *Ageing Res. Rev.* **92**, 102096 (2023). 37898293 10.1016/j.arr.2023.102096 PMC10824392

[249] Litzelman, K. Caregiver well-being and the quality of cancer care. *Semin. Oncol. Nurs.* **35**, 348–353 (2019). 31229346 10.1016/j.soncn.2019.06.006 PMC6728914

[250] Litzelman, K., Kent, E. E., Mollica, M. & Rowland, J. H. How does caregiver well-being relate to perceived quality of care in patients with cancer? Exploring associations and pathways. *J. Clin. Oncol.* **34**, 3554–3561 (2016). 27573657 10.1200/JCO.2016.67.3434 PMC5074348

[251] Fukushima, T. et al. Global quality of life and mortality risk in patients with cancer: a systematic review and meta-analysis. *Qual. Life Res.*10.1007/s11136-024-03691-3 (2024). 10.1007/s11136-024-03691-3 38811448

[252] Hill, C. V., Pérez-Stable, E. J., Anderson, N. A. & Bernard, M. A. The national institute on aging health disparities research framework. *Ethn. Dis.* **25**, 245–254 (2015). 26675362 10.18865/ed.25.3.245 PMC4671408

